# Neutrophil extracellular traps as a potential therapeutic target in inflammatory bowel disease: a systematic review and meta-analysis

**DOI:** 10.3389/fimmu.2026.1935261

**Published:** 2026-09-17

**Authors:** Yi-Jun Wu, Yao Huang, Hao Fang, Bing-Yan Gu, Hong-Cheng Ge, Ru-Yi Xu, Jia-Min Qian, Dan-Ping Qin

**Affiliations:** 1 The First Affiliated Hospital of Zhejiang Chinese Medical University (Zhejiang Provincial Hospital of Chinese Medicine), Hangzhou, China; 2 The First School of Clinical Medicine, Zhejiang Chinese Medical University, Hangzhou, Zhejiang, China; 3 Department of Nutrition, The First Affiliated Hospital of Zhejiang Chinese Medical University (Zhejang Provincal Hospital of Chinese Medicine), Hangzhou, Zhejiang, China; 4 Department of Gastroenterology, the First Affiliated Hospital of Zhejiang Chinese Medical University, Hangzhou, Zhejiang, China

**Keywords:** inflammatory bowel disease, meta-analysis, NETs modulation, neutrophil extracellular traps, preclinical model, targeted therapy

## Abstract

**Background:**

Elevated Neutrophil extracellular traps (NETs) associated biomarkers have been documented in inflammatory bowel disease (IBD) patients and preclinical models, but the therapeutic potential of targeting NETs remains unclear. This systematic review summarizes NETs-associated biomarkers in IBD patients and evaluates NETs-targeted interventions in preclinical models.

**Methods:**

Following PRISMA guidelines, we searched databases up to November 2025. Clinical and preclinical studies assessing NETs expression and targeted modulation in IBD were included. Standardized mean difference (SMD) and 95% confidence interval (CI) were calculated using random-effects models.

**Results:**

Forty-eight studies (12 clinical, 31 pre-clinical, 5 mixed) were identified. In clinical studies, NETs-associated biomarkers were elevated in the colonic mucosa and blood compared with healthy controls, and higher NETs burden was associated with more severe disease severity and poorer prognosis in IBD patients. In preclinical studies, direct NETs inhibition and degradation reduced disease activity index (SMD = -1.00 and -1.71, *P<* 0.05) and histological scores (SMD = -1.70 and -2.95, *P<* 0.01), and reversed colon shortening (*P<* 0.05). NETs modulation restored epithelial barrier by increasing occludin expression (SMD = 1.35 and 2.59, *P<* 0.01) and reducing intestinal permeability (*P<*0.05). Interventions suppressed pro-inflammatory cytokines (IL-1β, IL-6, TNF-α, and IFN-γ; *P<* 0.05) and increased anti-inflammatory cytokines (TGF-β, IL-10; *P<* 0.05). Subgroup analyses suggested administration route, modeling methods, and sample types may contribute to heterogeneity, although testing for subgroup differences was limited by the small number of studies. Sensitivity analyses indicated that pooled estimates for most outcomes were relatively stable, although several outcomes were not robust in sensitivity analyses after excluding individual studies. These findings should therefore be interpreted with caution.

**Conclusion:**

NETs accumulation is a hallmark pathological feature of IBD. In preclinical IBD models, targeted NETs modulation ameliorates colitis by restoring the intestinal barrier integrity and suppressing inflammatory cascades. Further high-quality preclinical and translational studies are needed to define standardized detection methods, optimal dosing, and delivery strategies for clinical translation.

**Systematic review registration:**

https://www.crd.york.ac.uk/PROSPERO, identifier CRD420261365119.

## 1 Introduction

Inflammatory bowel disease (IBD), including Ulcerative colitis (UC) and Crohn’s disease (CD), is a chronic relapsing disorder of the gastrointestinal tract. Patients present with abdominal pain, diarrhea, and bloody mucus (1, 2). IBD incidence continues to rise in newly industrialized countries but has stabilized in Western nations, making IBD a major global health burden (3). Current therapies include 5-aminosalicylates, corticosteroids, immunosuppressants, and biologics. However, long-term use increases the risk of opportunistic infections and hepatorenal toxicity (4). Moreover, 30–40% of patients have primary non-response to biologics, and another 10-15% lose response during maintenance therapy (5). Achieving sustained remission and mucosal healing remains challenging. New pathogenic mechanisms and therapeutic targets are urgently needed.

Neutrophil infiltration and crypt abscesses are hallmark features of active IBD (1). Neutrophils, key innate immune effectors, are recruited to inflamed sites by chemokines (6). Recent research has focused on NETosis, a form of programmed cell death that produces neutrophil extracellular traps (NETs). These structure traps and eliminate pathogens, but their excessive formation or impaired clearance disrupts intestinal homeostasis. In active UC, NETs-associated markers are elevated in the lamina propria and correlate with the endoscopic Mayo score. Neutrophil elastase (NE) within NETs can hydrolyze therapeutic antibodies (e.g., infliximab) into Fc monomers and IgG1 fragments, reducing their efficacy (7). Therefore, targeting NETs-associated pathological activity may offer a selective immunomodulatory strategy that attenuates mucosal injury while preserving host defense.

Current interventions include PAD4 inhibitors (e.g., Cl-amidine), PAD4 knockout, and DNase I (8, 9). Natural products and small molecules also inhibit NETs through multi-target mechanisms (10). Despite experimental evidence supporting NETs modulation in IBD, systematic quantitative evaluations are lacking due to heterogeneity in animal models, interventions, and administration routes. We therefore conducted this meta-analysis to synthesize two distinct lines of evidence: (1) observational clinical studies documenting NETs-associated biomarkers in IBD patients, and (2) interventional preclinical studies evaluating the efficacy of targeted NETs modulation in experimental colitis models. This study aims to determine whether NETs represent a viable therapeutic target in preclinical settings, and to identify knowledge gaps that need to be addressed before clinical translation.

## 2 Materials and methods

This meta-analysis was conducted following the Preferred Reporting Items for Systematic Reviews and Meta-Analyses (PRISMA) guidelines and was registered in the International Prospective Register of Systematic Reviews (PROSPERO) under registration number CRD420261365119.

### 2.1 Search strategy

We systematically searched PubMed, Embase, the Cochrane Library, Web of Science, and Scopus from database inception to November 21, 2025. The search strategy combined Medical Subject Headings (MeSH) terms, Emtree terms, and relevant keywords for “Inflammatory Bowel Disease” (including Crohn’s disease and Ulcerative colitis) and “Neutrophil Extracellular Traps”. No language restrictions were applied. A representative PubMed search string is provided in **Supplementary Table 1**.

### 2.2 Inclusion and exclusion criteria

Eligibility criteria were defined according to the PICOS (Participants, Intervention, Comparison, Outcome, and Study design) framework. The inclusion criteria are as follows: (1) Subjects (P): laboratory animal models of IBD with clearly described induction protocols. (2) Intervention and comparison (I/C): studies investigating the pathophysiological role of NETs in IBD or evaluating NETs-modulating therapies (including pharmacological inhibition, enzymatic degradation, or genetic ablation). Control groups had to receive vehicle treatments or no intervention. (3) Outcomes (O): primary outcomes included body weight changes, Disease Activity Index (DAI), colon length, histological injury scores, intestinal barrier integrity markers, and NETs-specific biomarkers. Secondary outcomes comprised pro-inflammatory cytokines and anti-inflammatory cytokines. (4) Study design (S): controlled preclinical studies, regardless of randomization status. We also included clinical studies that investigate the pathophysiological role of NETs in IBD patients.

The exclusion criteria are as follows: (1) editor letters, reviews, case reports, conferences, and extracellular cell line studies were excluded. (2) studies on non-related topics (those not discussing IBD and not studying NETs) were excluded. (3) studies with insufficient data or those where the required outcome measures could not be quantified or obtained from the authors. (4) articles not written in English or Chinese were excluded.

### 2.3 Study selection and data extraction

Literature screening was performed independently by two reviewers using EndNote 20 software. The selection followed a hierarchical process: (1) removal of duplicate records; (2) initial screening of titles and abstracts to exclude studies unrelated to IBD or NETs; (3) full-text assessment of the remaining articles against the predefined inclusion and exclusion criteria.

Data were extracted independently by two researchers to ensure accuracy. For preclinical studies, extracted parameters included first author, publication year, strain, sex, weight, age, dosage, frequency, route of administration, outcome measures, detection indicators, sample source, and detection methods. For clinical studies, we extracted data on the first author, year, study type, IBD subtype, sample size, sex, age, detection indicators, sample source, and detection methods.

For data presented only in graphs, numerical values were extracted using Engauge Digitizer. When data were missing or ambiguous, we contacted the corresponding authors by email. Disagreements during screening or extraction were resolved by consulting a third reviewer.

### 2.4 Risk of bias assessment

Two reviewers independently assessed methodological quality using the SYRCLE’s Risk of Bias (RoB) tool for animal studies. They rated each item as low, high, or unclear risk (11). For clinical studies, we used the Newcastle-Ottawa Scale (NOS) to evaluate the selection, comparability, and outcome of the case-control and cohort studies included (12). In addition, the Joanna Briggs Institute (JBI) critical appraisal was utilized to assess the risk of bias for cross-sectional studies (13). Disagreements were resolved through discussion or consultation with a third reviewer.

### 2.5 Statistical analysis

We extracted means, standard deviations (SD), and sample sizes (n) from each study. For data reported as standard error of the mean (SEM), we calculated $SD=\;SEM\times \sqrt{n}$. If a study included multiple intervention groups with different dosages, we pooled thier means and SDs into one composite group to avoid unit-of-analysis errors and double-counting of the control group. The treatment effect was estimated using the standardized mean difference (SMD) with 95% confidence interval (CI). Statistical heterogeneity across studies was assessed using the I^2^ statistic. A fixed-effects model was applied if heterogeneity was low (I^2^ ≤ 50%). Otherwise, a random-effects model was employed (I^2^ > 50%). To explore potential sources of significant heterogeneity, subgroup analyses were performed based on predefined variables. The robustness of the pooled estimates was further validated through leave-one-out sensitivity analysis. Publication bias was evaluated using funnel plots, supplemented by Egger’s and Begg’s tests when the number of included studies exceeded ten (n > 10). If publication bias was detected (*P* < 0.05), the trim-and-fill method was utilized to assess the stability of the results. All statistical analyses were conducted using Review Manager (version 5.4) and STATA/SE (version 15.1; StataCorp LLC, College Station, TX, USA).

## 3 Results

### 3.1 Study selection and characteristics

Our search identified 1, 134 records. After deduplication, 518 records were screened. 281 articles were excluded by title and abstract, and 237 underwent full-text review. Finally, 48 studies met the inclusion criteria. The screening process and exclusion reasons are presented in the PRISMA flowchart (**Figure 1**).

**Figure 1:**
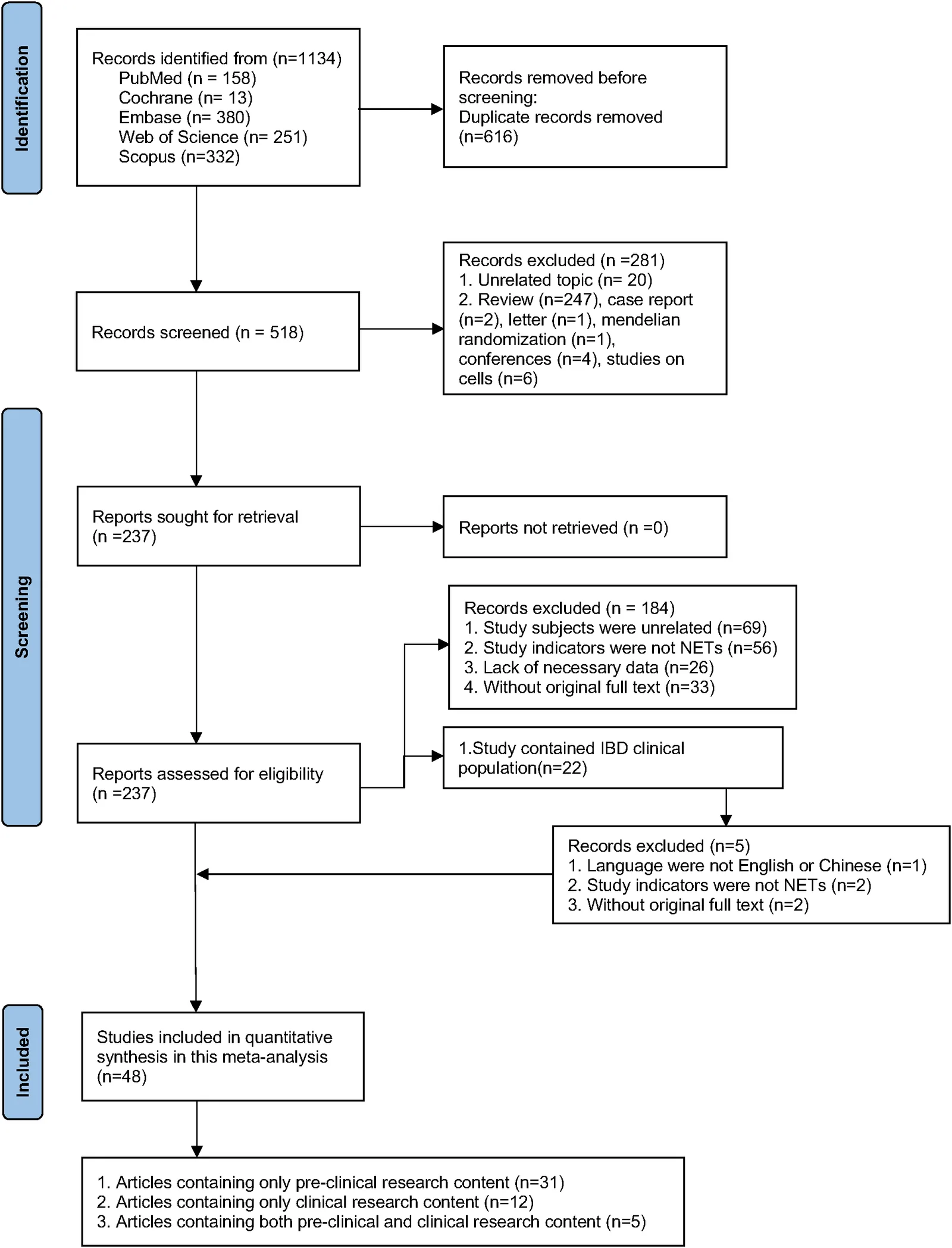
PRISMA flowchart showing the process of inclusion and exclusion.

A total of 48 studies, published between 2018 and 2025, were included. Among these, 31 articles contained only preclinical studies, 12 articles contained only clinical studies, and 5 articles included both preclinical and clinical components. Based on this classification, we analyzed the 17 clinical studies descriptively to characterize NETs-associated biomarker profiles in IBD patients. We subjected the 36 preclinical studies to quantitative meta-analysis to evaluate therapeutic efficacy.

Of the studies with preclinical content, 31 used mouse models: 25 used C57BL/6 mice, 5 used BALB/c mice, and 1 used KM mice. Five studies involved rat models, including three using Wistar rats and two using Sprague-Dawley rats. Regarding sex distribution, male animals were predominantly used (n=25). Six studies used only female animals, three studies included both sexes, and two studies did not specify sex. For IBD model induction, 28 studies used dextran sulfate sodium (DSS) exclusively, six used trinitrobenzene sulfonic acid (TNBS) alone, and one induced colitis with acetic acid. One additional study validated interventions in both DSS and TNBS models. Clinical and pathological assessments commonly included body weight change (29 studies), colon length (n=29), histological score (n=28), and disease activity index (DAI, n=28). Intestinal barrier function was specifically evaluated in 19 studies. Detection of NETs-associated biomarkers was comprehensive: citrullinated histone H3 (CitH3) was quantified in 31 studies, myeloperoxidase (MPO) in 28 studies, peptidylarginine deiminase 4 (PAD4) in 16 studies, and MPO-DNA complexes in 9 studies. Cytokine profiles were extensively reported. Among pro-inflammatory cytokines, tumor necrosis factor-alpha (TNF-α) was the most frequently measured (n=24), followed by interleukin-1β (IL-1β, n=22) and IL-6 (n=21). IL-17A and interferon-gamma (IFN-γ) were each quantified in six studies. For anti-inflammatory cytokines, IL-10 was measured in eight studies, and transforming growth factor-beta (TGF-β) in three studies. Regarding physiological baselines, mouse models showed relative consistency. Mouse ages ranged from 4 to 10 weeks, with 6–8 weeks being the most common. Initial body weights generally ranged from 18 to 27 g. Rat models exhibited greater weight variation (80–250 g), reflecting diverse experimental designs across laboratories. Detailed baseline characteristics of these studies are summarized in **Table 1**.

**Table 1:** Characteristics of the included preclinical studies in this meta-analysis.

Study	Year	Species	Sex	Weight(g)	Age(w)	Establishment of IBD models	Outcome index	NETs presence significantly confirmed (*P* < 0.05)	NETs locations	NETs marker	Method for detecting NETs
Almási et al. (69)	2023	Wistar Hannover Rat	male	150-175	/	TNBS, 10 mg/rat, 1 time, i.r.	(2)(11)(14)(15)(16)	Yes	Colon tissue	CitH3, MPO, PAD4	WB
Cao et al. (36)	2023	C57BL/6 mice	male	18-22	6-8	TNBS, 1.5 mg/mice, 1 time, i.r.	(1)(3)(4)(5)(7)(8)(10)(14)(15)(16)(17)(18)(20)(21)	Yes	Colon tissue	PAD4	IHC
										MPO, NE, CitH3	IF
										PAD4, MPO, NE, CitH3	WB
									Plasma	dsDNA	PicoGreen dsDNA Kit
										MPO-DNA	ELISA
									Serum	Ly6G	FCM
Dong et al. (39)	2021	C57BL/6 mice	female	/	8	DSS, 3% w/v, 7 days, drink	(1)(3)(4)(5)(6)(7)(8)(12)(16)(17)(21)	Yes	Colon tissue	NE, MPO, Ly6G	IHC
										NE, Ly6G	IF
Chen et al. (38)	2024	BalB/c mice	male	/	6-8	DSS, 5% w/v, 7 days, drink	(1)(3)(4)(5)(6)(10)(11)(12)(15)(16)	Yes	Colon tissue	MPO, CitH3	WB
										MPO, CitH3	IF
Ercan et al. (15)	2025	Wistar albino rat	male	200-250	8-10	Acetic acid, 4% w/v, 1 time, i.r.	(3)(10)(22)(23)(24)	Yes	Plasma	NETs (Unnamed specific markers)	ELISA
Lai et al. (16)	2023	C57BL/6 mice	Male and female	/	6	DSS, 3% w/v, 8 days, drink	(1)(3)(4)(5)(6)(7)(8)(9)(10)(13)(15)(16)	Yes	Colon tissue	MPO, CitH3	IF
Li et al. (66)	2024	C57BL/6 mice	male	18-22	/	DSS, 2.5% w/v, 7 days, drink	(1)(3)(5)(7)(8)(9)(10)(13)(15)(16)	Yes	Colon tissue	MPO, CitH3	IF
Lin et al. (14)	2020	C57BL/6 mice	female	/	7	DSS, 2.5% w/v, 8 days, drink	(1)(3)(4)(5)(6)(7)(9)(10)(15)(16)(17)(18)	Yes	Colon tissue	MPO, CitH3, NE	IF
						TNBS, 2.5% w/v, 1 time, i.r.	(3)(4)(5)(6)(7)(9)(10)(15)(16)(17)(18)			MPO-DNA	ELISA
Liu et al. (70)	2025	BalB/c mice	Male	/	8-10	DSS, 3.5% w/v, 7 days, drink	(1)(3)(4)(5)(6)(7)(8)(16)	Yes	Colon tissue	MPO	ELISA
Chi et al. (17)	2024	Sprague-Dawley Rat	Male	4	80-120	TNBS 5%+50% ethanol, 4 weeks, i.r.,	(15)(16)(17)(18)	Yes	Colon tissue	MPO, NE, CitH3	IF、RT-qPCR
									Plasma	MPO-DNA, NE-DNA	ELISA
Ma et al. (18)	2024	C57BL/6 mice	Male	6-8	18-20	DSS, 3.0% w/v, 7 days, drink	(1)(3)(4)(5)(6) (7)(8)(10)(13) (14)(15)(17) (19)(21)	Yes	Colon tissue	NE, CitH3, PAD4	WB
										CitH3, Ly6G	IF
									Plasma	Cf-DNA	ELISA
Qin et al. (71)	2024	C57BL/6J mice	Male	4-8	/	DSS, 2.0% w/v, 7 days, drink	(1)(3)(4)(5)(6)(7)(8)(10)(14)(15)(16)	Yes	Colon tissue	Ly6G, CitH3	IF
										MPO, PAD4, CitH3	WB
Sun et al. (72)	2024	C57BL/6 mice	Male and female	6-8	20-25	Circle 1: DSS, 2.5% w/v, 5 days, distilled H_2_O 7 days, drink Circle 2: DSS, 2.5% w/v, 5 days, distilled H_2_O, 5 days, drink	(1)(3)(4)(5)(6)(7)(8)(10)(14)(15)(16)(18)	Yes	Colon tissue	PAD4, CitH3	WB
									Plasma	MPO-DNA, CitH3	ELISA
Tang et al. (73)	2024	C57BL/6 mice	Male	6-8	/	DSS, 2.0% w/v, 7 days, drink	(1)(3)(4)(5)(7)(8)(10)(14)(15)(16)	Yes	Colon tissue	PAD4	WB
									Plasma	MPO, CitH3	ELISA
Török et al. (74)	2021	Wistar–Harlan Rat	Male	/	225–250	TNBS, 50%ethanol, 1 time, i.r.,	(2)(14)(15)(16)	Yes	Colon tissue	PAD4, MPO, CitH3	WB
Wang et al. (75)	2023	C57BL/6 mice	Male and female	6-8	20-25	DSS, 3.0% w/v, 7 days, drink Circle 1: DSS, 2.5% w/v, 5 days, distilled H_2_O 7 days, drink Circle 2: DSS, 2.5% w/v, 5 days, distilled H_2_O, 5 days, drink	(1)(3)(4)(5)(6)(7)(8)(14)(15)(16)(18)	Yes	Colon tissue	PAD4, MPO, CitH3	WB
Wang et al. (76)	2025	C57BL/6 mice	Male	6-8	22-25	DSS, 2.5% w/v, 7 days + normal water, 3 days, drink	(1)(3)(4)(5)(6)(8)(10)(13)(14)(15)(16)(17)	Yes	Colon tissue	PAD4	WB
										MPO	ELISA
										PAD4, MPO, CitH3, NE	IF
Wei et al. (64)	2025	C57BL/6 mice	Male	7-8	18-20	DSS, 2.5% w/v, 7 days + normal water, 2 days, drink	(1)(3)(4)(5)(6)(8)(9)(10)(12)(14)(15)(16)(17)(21)	Yes	Colon tissue	MPO, CitH3, PAD4	IHC
										CitH3, NE	IF
Wang et al. (6)	2024	KM mice	Female	6-8	18–20	DSS, 3.0% w/v, 7 days, drink	(1)(3)(4)(5)(6)(7)(8)(10)(13)(14)(15)(16)(17)	Yes	Colon tissue	MPO, PAD4, CitH3	WB
										MPO, PAD4	IHC
										MPO, CitH3, DNA	IF
									Feces	MPO	ELISA
Wen et al. (77)	2022	Sprague-DawleyRat	Male	8-12	225-250	DSS, 3% w/v, 7 days, drink	(1)(3)(7)(10)(14)(15)(16)(17)	Yes	Colon tissue	PAD4, CitH3, MPO, NE	WB
Xie et al. (37)	2025	C57BL/6 mice	Male	6	/	DSS, 2.0% w/v, 9 days, drink	(1)(3)(4)(5)(6) (7)(13)(15)(16) (17)	Yes	Colon tissue	MPO, NE, CitH3	IF
Xu et al. (34)	2023	C57BL/6 mice	Male	7-8	18–20	DSS, 3.0% w/v, 7 days, drink	(1)(3)(4)(5)(7)(10)(15)(17)	Yes	Colon tissue	NE, CitH3	IF
											WB
Yang et al. (1) (78)	2025	C57BL/6 mice	Male	6	/	DSS, 2.5% w/v, 8 days, drink	(1)(2)(3)(4)(5)(15)(16)(21)	Yes	Colon tissue	MPO, CitH3	IF
Yang et al. (2) (79)	2025	BALB/c mice	Male	6	/	DSS, 2.5% w/v, 8 days, drink	(1)(3)(4)(5)(15)(16)	Yes	Colon tissue	MPO, CitH3	IF
Yang et al. (80)	2024	C57BL/6 mice	Male	6-8	18-22	DSS, 2.5% w/v, 7 days, drink	(1)(4)(5)(6)(7)(8)(10)(14)(19)(15)(16)	Yes	Colon tissue	MPO, CitH3	IF
										PADI4, MPO, ELANE	qPCR
									Serum	cfDNA	ELISA
Yasuda et al. (81)	2024	C57BL/6 mice	Male	8-9	21-27	TNBS, 0.1 mL, once time, i.r.,	(4)(7)(10)(14) (15)(16)	Yes	Colon tissue	CitH3, MPO	IF
Ye et al. (82)	2025	C57BL/6 mice	Male	8	/	DSS, 2.5% w/v, 7 days, drink	(1)(4)(5)(6)(7)(8)(10)(15)(21)	Yes	Colon tissue	CitH3, Ly6G	IF
										CitH3	WB
									Serum	MPO	ELISA
Zhang et al. (83)	2025	C57BL/6 mice	/	7	/	DSS, 3.0% w/v, 7 days, drink	(1)(3)(4)(5)(6)(7)(8)(10)(11)(14)(15)(16)	Yes	Colon tissue	MPO, NE, PAD4	IF
										MPO, CitH3, PAD4	WB
Zhang et al. (65)	2023	BALB/c mice	Female	8	/	DSS, 3.0% w/v, 7 days, drink	(18)	Yes	Plasma	MPO-DNA	ELISA
Zhang et al. (84)	2020	BALB/c mice	Female	8-10	/	2.5% TNBS in 50% ethanol, once time, i.r.,	(1)(3)(4)(5)(7)(8)(9)(10)(12)(13)(14)(15)(16)(21)	Yes	Colon tissue	CitH3, Ly6G	IHC
										PAD4	qPCR
Zhu et al. (85)	2025	C57BL/6 mice	Male	6	/	DSS, 3.0% w/v, 7 days, drink	(1)(3)(4)(5)(6)(14)(15)(16)(18)	Yes	Colon tissue	MPO, CitH3	IHC
											WB
									Plasma	MPO-DNA	ELISA
Zhu et al. (86)	2023	C57BL/6 mice	/	8-10	20-25	DSS, 2.5% w/v, 7 days, drink	(1)(3)(5)(7)(8)(10)(12)(15)	Yes	Colon tissue	CitH3, Ly6G	IHC
										CitH3	WB
										MPO	qPCR
Li et al. (22)	2020	C57BL/6 mice	Male	8-10	/	DSS, 3.5% w/v, 6 days, drink	(1)(3)(4)(5)(6)(7)(8)(10)(15)(18)(19)	Yes	Plasma	Cf-DNA, MPO-DNA	ELISA
									Colon tissue	CitH3, NE, Ly6G	IF
										CitH3	WB
Otsuka et al. (35)	2023	C57BL/6 mice	Female	6	/	DSS, 2.0% w/v, 5 days, drink	(3)(4)(5)	/	/	/	/
Shao et al. (28)	2023	C57BL/6 mice	Male	8	/	DSS, 2.0% w/v, 7 days, drink	(1)(4)(5)(6)(8)(9)(10)(13)(15)(16)(18)	Yes	Colon tissue	CitH3, MPO	IF
									Plasma	MPO-DNA, MPO	ELISA
Li et al. (87)	2021	C57BL/6 mice	Male	8-10	20-25	DSS, 2.0% w/v, 7 days, drink, normal water, 3 day, drink	(3)(4)(5)(8)(10)(12)(15) (21)	Yes	Colon tissue	CitH3, Ly6G	IF
										CitH3	WB

Outcome measure: (1) DAI, (2) Macroscopic pathological score, (3) Histological score, (4) Body weight, (5) Colon length, (6) Intestinal barrier function, (7) interleukin (IL)-1β, (8) IL-6, (9) IL-17A, (10) Tumor necrosis factor (TNF)-α, (11) Transforming growth factor (TGF)-β, (12) IFN-γ, (13) IL-10, (14) Peptidylarginine deiminase 4 (PAD4), (15) Citrullinated histone H3 (CitH3), (16) Myeloperoxidase (MPO), (17) Neutrophil elastase (NE), (18) MPO-DNA (19) Cell-free DNA (cf-DNA), (20) Double-stranded DNA (ds-DNA) (21) Lymphocyte antigen 6 complex locus G6D (Ly6G), (22) Pentraxin 3 (PTX3), (23) Vascular endothelial growth factor (VEGF), (24) Malondialdehyde (MDA).

Detection methods: Western Blot (WB), Immunohistochemistry (IHC), Immunofluorescence (IF), Enzyme-Linked Immunosorbent Assays (ELISA), quantitative PCR (qPCR), Flow cytometry (FCM).

A total of 17 clinical observational studies published between 2018 and 2025 were included to evaluate the role of NETs in human IBD. These primarily consisted of case-control (n = 12), cross-sectional (n = 3), and retrospective cohort designs (n = 2), covering adult and pediatric patients with UC and CD, including phenotypes such as perianal fistulizing CD. NETs burden was assessed by comparing active IBD with healthy controls or remission states. Beyond marker expression, these studies analyzed the correlation between NETs abundance and clinical parameters, including disease activity, pro-inflammatory cytokines, treatment response, and prognosis. Specimens included colonic mucosal biopsies, peripheral blood (plasma/serum), and feces. Core markers analyzed were CitH3, PAD4, MPO, NE, cf-DNA, and MPO-DNA complexes. Detailed characteristics of these studies are summarized in **Table 2**.

**Table 2:** Characteristics of the included clinical studies in this meta-analysis.

Study	Year	Study design	IBD subtype				Control group				NETs presence significantly confirmed (compared with NC, *P* < 0.05)	The locations where NETs exist	NETs marker	Method for detecting NETs	Main clinical findings
				Number	Age	Sex (male%)	Control type	Number	Age	Sex (male%)					
El Hafez et al. (20)	2020	Retrospective cohort study	UC	N = 42	39.4 ± 13.8	52.4%	NC	N = 11	41.6 ± 14.7	54.5%	Yes	Colon tissue	PAD4	IHC	PAD4 expression significantly differs between UC and normal colon, positively correlates with histopathological activity grade, and is associated with therapy response and radical surgery.
Cao et al. (25)	2024	Cross-sectional study	pfCD (NETs-positive)	N = 19	/	84.2%	pfCD (NETs-negative)	N = 21	/	76.2%	Yes	Perianal fistula	CitH3	ELISA	NETs correlate negatively with tissue IFX levels and fistula healing.
												Perianal fistula	CitH3, MPO	IF	
Cao et al. (19)	2023	Retrospective cohort study	pfCD (unhealed)	N = 10	/	60%	pfCD (unhealed)	N = 11	/	90.9%	Yes	Perianal fistula	PAD4, NE;	IHC	(1) In pfCD, NETs are present in fistulas and correlate inversely with healing. (2) Fistula NETs levels correlate negatively with local IFX concentrations.
													MPO, NE, CitH3	IF	
													MPO, CitH3	ELISA	
													PAD4	qPCR	
Angelidou et al. (32)	2018	Case-control study	UC	N = 15	38.5 ± 17.4	66.7%	NC	N = 25	37.8 ± 13.3	52%	Yes	Colon tissue	NE, CitH3	IF	UC patients have higher colonic NETs accumulation than CD patients and healthy controls.
			CD	N = 11	36.7 ± 19.4	63.6%									
Dinallo et al. (29)	2019	Case-control study	UC	N = 9	/	/	NC	N = 12	/	/	Yes (for UC patients)	Colon tissue	PAD4	IHC	NETs-associated proteins are highly expressed in the colon of UC patients.
			CD	N = 9	/	/							PAD4, MPO, NE, CitH3	WB	
			UC (non-inflamed)	N = 4	/	/	UC (inflamed)	N = 4	/	/			MPO, NE, CitH3	IF	In UC patients, PAD4 expression is higher in inflamed sites than in their own non-inflamed sites.
Drury et al. (26)	2023	Case-control study	IBD	N = 31	/	48.4%	NC	N = 10	/	/	No	Plasma	CitH3	ELISA	(1) Plasma CitH3 levels do not differ between active IBD patients and healthy controls. (2) Active IBD patients have lower fecal CitH3 levels than those in remission. (3) Fecal CitH3 correlates negatively with calprotectin.
												Fecal supernatant	CitH3	ELISA	
Gottlieb et al. (30)	2018	Case-control study	UC	N = 6	/	33.3%	NC	N = 2	/	/	Yes	Colon tissue	NE, MPO	IF	NETs are present in biopsies from CD and UC patients, but not in healthy controls.
			CD	N = 6	/	50%									
Kong et al. (24)	2025	Cross-sectional study	UC	N = 36	8.3 ± 5.0	63%	NC	N = 20	10.1 ± 3.5	30%	Yes	Serum	NE, MPO-DNA	ELISA	(1) Children with UC and CD have higher peripheral blood NE and MPO-DNA levels than healthy children; (2) In children with IBD, NETs markers do not correlate with clinical or endoscopic activity.
			CD	N = 30	11.0 ± 3.7	64%									
Lehmann et al. (21)	2019	Cross-sectional study	UC	N = 14	/	/	NC	N = 17	/	/	Yes	Feces	NE, MPO, azurocidin, cathepsin G, myeloblastin, PADI2, S100A8/A9	Metaproteomic	Fecal NE and MPO (NETs-related proteins) are upregulated in IBD patients.
			CD	N = 11	/	/									
Li et al. (22)	2020	Case-control study	UC	N = 24	/	/	NC	N = 10	44.2 ± 14.4	40%	Yes	Plasma	Cf-DNA	Fluorescence quantitative	(1) Enhanced NETs release in IBD patients;(2) NETs deposition in the colon of IBD patients; (3) Impaired NETs degradation in the plasma of IBD patients.
			CD	N = 24	/	/							MPO-DNA	ELISA	
												Colon tissue	NE, CitH3	IF	
Lu et al. (23)	2025	Case-control study	IBD-active	N = 36	49.35 ± 11.05	18%	NC	N = 100	49.25 ± 14.10	58%	Yes	Plasma	Cf-DNA,	Fluorescence quantitative	(1) Active IBD patients have higher circulating NETs marker levels than inactive patients. (2) Circulating NETs marker levels correlate positively with inflammatory factors. (3) A high NETs score is a poor prognostic factor for IBD relapse.
												Plasma	MPO-DNA, CitH3	ELISA	
			IBD-inactive	N = 64	48.24 ± 14.35	34%									
Otsuka et al. (35)	2023	Case-control study	UC-active	N = 20	/	/	NC	N = 4	/	/	NO	Colon tissue	PAD4, PAD2	qpCR	(1) Active UC patients have higher colonic mucosal PAD4 but lower PAD2 mRNA than those in remission, with a significant negative correlation between the two;(2) In UC patients, mucosal PAD4 mRNA levels correlate positively with disease activity indices and pro-inflammatory cytokine profiles.
			UC-remission	N = 20	/	/									
			CD-active	N = 10	/	/									(1) Colonic PAD4 and PAD2 mRNA levels do not differ between active and remission phases of CD. (2) Mucosal PAD4 mRNA levels correlate positively with pro-inflammatory cytokine profiles in CD patients.
			CD-remission	N = 10	/	/									
Schoen et al. (27)	2024	Case-control study	UC	N = 12	/	/	NC	N = 96	/	/	Yes	Serum	cf-DNA, NE-DNA, MPO-DNA	Fluorescence quantitative	(1) UC and CD patients have higher serum cf-DNA levels than healthy controls; (2) CD patients show lower NE activity than healthy controls.
			CD	N = 37	/	/							CitH3	ELISA	
Schroder et al. (31)	2022	Case-control study	CD	N = 6	43.3 ± 23.3	83%	NC	N = 3	65.3 ± 9.0	66.6%	Yes	Colon tissue	MPO, NE, CitH3	IF	(1) MPO and NE staining is stronger in CD lesional and transitional tissues than in uninvolved areas or healthy controls;(2) Immunostaining for MPO, NE, and CitH3 shows a significant linear increase with CD histopathological severity.
Shao et al. (28)	2023	Case-control study	IBD	/	/	/	NC	/	/	/	Yes	Plasma	CitH3	ELISA	IBD patients have elevated blood CitH3 levels and colonic NETs deposition.
												Colon tissue	MPO, CitH3	IF	
Shukrun et al. (33)	2024	Case-control study	UC	N = 9	/	33.3%	NC	N = 9	/	45%	Yes	Colon tissue	NE, CitH3	IF	In pediatric IBD patients, intestinal NETs formation is elevated versus controls, and inflamed mucosa shows a higher NETs burden than uninvolved areas.
			CD	N =11	/	63.6%									
Xu et al. (34)	2023	Case-control study	UC	NC = 6	/	/	NC	N=6	/	/	Yes	Colon tissue	NE, CitH3	IF	(1) UC patients have higher colonic mucosal NE and CitH3 than healthy controls. (2) NETs enrichment scores increase with UC severity and are significantly higher in moderate-to-severe patients than in mild cases or healthy individuals.

Citrullinated Histone H3 (CitH3), Myeloperoxidase (MPO), Neutrophil Elastase (NE), Peptidylarginine deiminase 4 (PAD4), cell-free DNA (cf-DNA), double-stranded DNA (ds-DNA),  lymphocyte antigen 6 complex locus G6D (Ly6G), Western Blot (WB), Immunohistochemistry (IHC), Immunofluorescence (IF), Enzyme-Linked Immunosorbent Assays (ELISA), quantitative PCR (qPCR), Flow cytometry (FCM).

### 3.2 NETs modulation

We identified three categories of NETs modulation strategies in the included studies: direct inhibition, direct degradation, and indirect modulation. Direct inhibition was achieved through genetic ablation or pharmacological blockade of the two key enzymatic drivers, PAD4 and MPO. PAD4 was selectively targeted by Cl-amidine or GSK484, while MPO was targeted by AZD3241. Cl-amidine and GSK484 were given mostly by daily intraperitoneal injection or oral gavage, whereas AZD3241 was administered via gavage. TP5, a thymopentin derivative, was delivered subcutaneously in one study. Direct degradation involves DNase I or ALG-SNase to cleave extracellular DNA networks, administered mainly via intraperitoneal or intravenous injection. Several studies used PAD4 knockout mice, providing genetic validation of the pathological role of NETs in IBD. By contrast, indirect modulation encompasses a diverse range of interventions without specific NETs-directed mechanisms, including natural compounds (e.g., quercetin, berberine, and dihydromyricetin), traditional Chinese medicine formulations (e.g., Da-yuan-yin and Kui-yang-ling), and biological therapies (e.g., Bacteroides fragilis-derived outer membrane vesicles and Coptis chinensis-derived extracellular vesicle-like nanoparticles). Some studies also used physical interventions such as exercise and moxibustion. In our quantitative meta-analysis, we included only the direct inhibition and direct degradation studies, as these provide interpretable evidence for assessing whether NETs are sufficient to drive colitis pathology. Across these direct interventions, both degradation and inhibition strategies ameliorated experimental colitis, as reflected by reduced body weight loss, colon shortening, DAI, and histological scores, along with restored intestinal barrier integrity and corrected cytokine imbalance. However, results were not entirely consistent. One study reported that isolated PAD4 or MPO inhibitors failed to improve clinical parameters, barrier function, or inflammatory responses. Indirect interventions were summarized descriptively in **Table 3** but were not included in the pooled effect size calculations, as their pleiotropic mechanisms could confound attribution of therapeutic benefit specifically to NETs modulation. Detailed strategies are in **Table 3**.

**Table 3:** The NETs modulation strategy of the included studies.

Study	Intervention category	Specific intervention	Dosing strategy	Outcomes after intervention
Almási et al. (69)	Indirect modulation	voluntary exercise	Pre-administer 6 weeks of voluntary exercise before the modeling process	A 6-week voluntary exercise program can alleviate colonic inflammation induced by TNBS, reduce the levels of NETosis-related markers, and increase the level of the antioxidant enzyme Prdx6 (*P* < 0.05 for all results).
Cao et al. (36)	Direct inhibition	TP5	25 mg/kg, s.c., 7 days	TP5 treatment significantly attenuated body weight loss and colon shortening, while reducing DAI scores and histological damage in mice with colitis. Moreover, it decreased the levels of pro-inflammatory cytokines and downregulated the expression of NETs-related markers (*P* < 0.05 for all results).
Dong et al. (39)	Direct degradation	ALG-SNase	25 or 75 mg/kg, i.g., 7 days	ALG-SNase and Cl-amidine significantly increased survival rates and attenuated disease progression, characterized by preserved body weight and colon length, as well as reduced DAI and histopathological scores. Furthermore, both agents restored intestinal barrier integrity, inhibited colonic NETs formation, and suppressed pro-inflammatory cytokine levels in the colon and peripheral blood (*P* < 0.05 for all results).
	Direct inhibition	Cl-amidine	25 mg/kg, i.g., 7 days	
Chen et al. (38)	Indirect modulation	Gly	50 mg/kg, i.p., 7 days	Both monotherapy and combined therapy with Gly and DNase I significantly attenuated disease severity, evidenced by preserved colon length and body weight, as well as decreased DAI and histological damage. These interventions restored intestinal barrier function (ZO-1, occludin), inhibited NETs formation, and reduced systemic and local pro-inflammatory cytokine levels, facilitating an M1-to-M2 macrophage transition. Gly significantly inhibited the expression of HMGB1 as well as the receptors TLR2, TLR4 and RAGE (*P* < 0.05 for all results).
	Direct degradation	DNase I	65 U/mice, i.v., 7 days	
Ercan et al. (15)	Indirect modulation	Suramin	10 mg/kg, i.p., 15 days	Suramin significantly decreased plasma NETs levels in rats with acute colitis. Moreover, it reduced plasma levels of the pro-inflammatory cytokine TNF-α, the acute-phase protein PTX3, and the oxidative stress marker MDA, while downregulating local colonic TNF-α and VEGF and ameliorating histopathological scores (*P* < 0.05 for all results).
Lai et al. (16)	Direct inhibition	PAD4 knockout	/	NETs modulation significantly attenuated body weight loss reduced clinical and pathological scores, and preserved colon length. PAD4 deficiency downregulated colonic pro-inflammatory cytokines (IL-1β, IL-6, TNF-α), decreased intestinal permeability and bacterial translocation, and upregulated the expression of the tight junction protein ZO-1 (*P* < 0.05 for all results).
Li et al. (66)	Indirect modulation	Kui-Jie-Lin	1.5 g or 3.0 g/kg, i.g., 8 days	Kujieling capsules dose-dependently ameliorated colitis by reducing DAI and histopathological scores, while preserving colon length and body weight. The treatment rebalanced cytokine levels (downregulating pro-inflammatory and upregulating anti-inflammatory cytokines) and concurrently inhibited the LPS–TLR4/NF-κB and IL-23–JAK2/STAT3 signaling pathways, as well as NETs formation (*P* < 0.05 for all results).
Lin et al. (14)	Direct degradation	DNase I	250 U/mice, i.v., 7 days	In DSS group, DNase I degradation of NETs significantly ameliorated disease severity, preserved body weight and colon length, along with decreased DAI and histological scores. It reduced pro-inflammatory cytokine levels and restored the barrier function of the colonic epithelium (*P* < 0.05 for all results).
			250 U/mice, i.v., 3 days	In TNBS group, DNase I degradation of NETs significantly ameliorated disease severity, preserved body weight and colon length, along with decreased histological scores. It reduced pro-inflammatory cytokine levels and restored the barrier function of the colonic epithelium (*P* < 0.05 for all results).
Liu et al. (70)	Indirect modulation	Lactobacillus johnsonii N5	10^8^ CFU/mice, i.g., 14 days	In N5+DSS group, modulation preserved colon length (p<0.05), along with decreased histological scores, reduced pro-inflammatory cytokine levels (IL-1β and IL-6) and restored mucus barrier function (*P* < 0.05 for all results).
			10^8^ CFU/mice, i.g., 7 days	In DSS+N5 group, modulation restored body weight, preserved colon length, reduced pro-inflammatory cytokine levels (IL-1β, IL-6, and IL-8), improved intestinal barrier function and reduced the formation of NETs (*P* < 0.05 for all results).
	Direct degradation	DNase I	10 U/mice, i.p., 4 days	In DNase I group, degradation of NETs significantly mitigated body weight loss (*P* < 0.01), showed a tendency to reduce DAI (*P* = 0.06), reduced the number of CitH3+ neutrophils in blood (*P* < 0.05).
Chi et al. (17)	Indirect modulation	Moxibustion	moxibustion at CV6 (Qihai) and ST25 (Tianshu), 90 mg/2 zhuang/mice, 7 days	Moxibustion significantly reduced the CMDI score and lowered serum levels of NETs-DNA, NE-DNA, and MPO-DNA. Additionally, it downregulated colonic NE, CitH3, and MPO expression (*P* < 0.05 for all results).
Ma et al. (18)	Indirect modulation	DHM	50 or 100 mg/kg, i.g., 7 days	Modulation of NETs significantly ameliorated body weight loss and colon shortening, reduced DAI and histopathological scores, restored intestinal barrier integrity and suppressed pro-inflammatory cytokines (IL-1β, IL-6 and TNF-α) in both the colon and serum (*P* < 0.05 for all results).
Qin et al. (71)	Indirect modulation	Arbutin	50 or 100 mg/kg, i.g., 28 days	NETs modulation significantly improved DAI, histological scores, body weight, colon length, restored colon barrier function, and reduced the levels of inflammatory factors (IL-1β, IL-6, TNF-α, KC and CCL2) and neutrophil recruitment in colon tissues (*P* < 0.05 for all results).
Sun et al. (72)	Direct inhibition	PAD4 knockout	/	NETs modulation significantly improved DAI, histopathological scores, body weight, colon length, restored intestinal barrier function, and reduced serum pro-inflammatory factor levels (TNF-α, IFN-β) (*P* < 0.05 for all results).
Tang et al. (73)	Indirect modulation	Berberine	10 or 20 mg/kg, i.g., 7 days	NETs modulation significantly preserved body weight and colon length, reduced DAI and histological score, and downregulated colonic pro-inflammatory cytokines (IL-1β, IL-6, TNF-α) (*P* < 0.05 for all results).
	Direct inhibition	GSK484	4 mg/kg, i.g., 7 days	
Török et al. (74)	Indirect modulation	Hydrogen sulfide (H2S)	18.75 μmol/kg, i.g., 3 days	NETs modulation significantly improved the macroscopic injury indicators of the colon.
Wang et al. (75)	Direct inhibition	Cl-amidine	25 mg/kg, i.g., 7 days	NETs modulation significantly preserved body weight and colon length, reduced DAI and histological score, downregulated colonic pro-inflammatory cytokines and reestablished immune dysregulation (*P* < 0.05 for all results).
	Direct inhibition	PAD4 knockout	/	
Wang et al. (76)	Indirect modulation	Forsythiaside A	15 mg or 30 mg or 60 mg/kg, i.g., 10 days	NETs modulation significantly preserved body weight and colon length, reduced DAI and histological score, downregulated colonic pro-inflammatory cytokines (IL-6, TNF-α), upregulated anti-inflammatory cytokines (IL-10) (*P* < 0.05 for all results).
Wei et al. (64)	Indirect modulation	Quercetin	20 or 50 mg/kg, i.g., 9 days	NETs modulation significantly preserved body weight and colon length, reduced DAI and histological score, downregulated pro-inflammatory cytokines (TNF-α, IFN-γ, IL-6 and IL-17a), and inhibited oxidative stress (*P* < 0.05 for all results).
Wang et al. (6)	Indirect modulation	Pulsatilla Decoction	20 or 40 or 80 mg/kg, i.g., 7 days	NETs modulation significantly preserved body weight and colon length, reduced DAI and histological score, downregulated colonic pro-inflammatory cytokines (IL-1β, IL-6, TNF-α), upregulated anti-inflammatory cytokines (IL-4 and IL-10), inhibited neutrophil infiltration and activation (*P* < 0.05 for all results).
Wen et al. (77)	Indirect modulation	Anti-FcRn monoclonal antibody	80 or 160 μg/kg, i.v., 8 days	NETs modulation significantly reduced DAI and histological score, and downregulated serum pro-inflammatory cytokines (TNF-α, IL-1β, and CRP) (*P* < 0.05 for all results).
Xie et al. (37)	Direct inhibition	GSK484	4 mg/kg, i.p., 8 days	Following NETs intervention, no significant amelioration was observed in key disease indices, including body weight, DAI scores, histopathological damage, or colon length. Likewise, intestinal barrier integrity and colonic inflammatory cytokine levels remained unimproved (*P*>0.05 for all results).
		AZD3241	30 mg/kg, i.g., 9 days	
		GSK484+AZD3241	GSK: 4 mg/kg, i.p., 8 days AZD: 30 mg/kg, i.g., 9 days	
Xu et al. (34)	Indirect modulation	Cyclosporine A	CsA, i.p., 7 days	NETs modulation significantly preserved body weight and colon length, reduced DAI and histological scores, and downregulated pro-inflammatory cytokine (IL-1β and TNF-α) (*P* < 0.05 for all results).
Yang et al. (1) (78)	Indirect modulation	Cc-ELNs	50 μg/d, i.p., 8 days	NETs modulation significantly preserved body weight and colon length, reduced DAI and histological scores (*P* < 0.05 for all results).
	Direct degradation	DNase I	20 mg/kg, i.p., 8 days	
Yang et al. (2) (79)	Indirect modulation	Bf-OMVs	2×10^10^ OMVs, i.g., 8 days 50 μg Bf-OMVs, qd, i.p., 8 days	NETs modulation significantly preserved body weight and colon length, reduced DAI and histological scores (*P* < 0.05 for all results).
Yang et al. (80)	Indirect modulation	Da-yuan-yin decoction	0.4 or 0.8 or 1.6 g/kg, i.g., 7 days	NETs modulation significantly preserved body weight and colon length, reduced DAI and histological scores, downregulated pro-inflammatory cytokine (TNF-α, IL-1β, IL-6, IL-8, IL-17), upregulated anti-inflammatory cytokine (IL-10) and improved the function of the mucosal barrier (*P* < 0.05 for all results).
Yasuda et al. (81)	Direct inhibition	Cl-amidine	30 mg/kg, i.p., 3 days	NETs modulation significantly preserved body weight (DNase I and PAD4 knockout) downregulated the level of IL-1β (DNase I, Cl-amidine and PAD4 knockout), TNF-α (DNase I and PAD4 knockout) (*P* < 0.05 for all results).
	Direct degradation	DNase I	0.4 mg/kg, i.v. + 2 mg/kg, i.p., 3 days	
		PAD4 knockout	/	
Ye et al. (82)	Indirect modulation	Oat Avenanthramide-C	5 mg or 30 mg/kg, i.g., 14 days	NETs modulation significantly preserved body weight and colon length, reduced DAI scores, protect mucosal barrier and downregulated pro-inflammatory cytokines (TNF-α, IL-17, IL-1β, IL-6) (*P* < 0.05 for all results).
Zhang et al. (83)	Indirect modulation	Kuiyangling Enema	5 or 10g/kg, p.r., 7 days	NETs modulation significantly preserved body weight and colon length, reduced DAI and histological scores, protect mucosal barrier, downregulated pro-inflammatory cytokine (IL-1β, IL-6, TNF-α) and upregulated anti-inflammatory cytokine (TGF-β, IL-10, IL-37) (*P* < 0.05 for all results).
Zhang et al. (65)	Indirect modulation	Berberine	1 mg/kg, i.p., 7 days	NETs modulation significantly reduced level of MPO-DNA in serum (*P* < 0.05).
	Direct degradation	DNase I	0.1 U/mice, i.v., 7 days	
	Indirect modulation	GW4869	2.5 mg/Kg, i.p., 7 days	
Zhang et al. (84)	Direct inhibition	Cl-amidine	30 mg/kg, i.p., 6 days	NETs modulation significantly preserved colon length, reduced histological score, downregulated pro-inflammatory cytokine (IL-1β, IL-6, TNF-α) (*P* < 0.05 for all results).
Zhu et al. (85)	Indirect modulation	exercise intervention	/	NETs modulation significantly preserved body weight and colon length, reduced DAI and histological scores, impaired intestinal mucosal barrier (*P* < 0.05 for all results).
	Direct inhibition	Cl-amidine	25 mg/kg, i.p., 5 days	
Zhu et al. (86)	Indirect modulation	Syk inhibitor R788	30 mg/kg, i.g., 7 days	NETs modulation significantly preserved colon length, reduced DAI and histological scores, and downregulated pro-inflammatory cytokines (IL-1β, IL-6, TNF-α, IFN-γ) (*P* < 0.05 for all results).
Li et al. (22)	Direct degradation	DNase I	65 U/mice, i.v., 8 days	NETs modulation significantly preserved body weight and colon length, reduced DAI and histological scores, impaired intestinal mucosal barrier, and downregulated pro-inflammatory cytokines (IL-1β, IL-6, TNF-α) (*P* < 0.05 for all results).
	Indirect modulation	Anti-Ly6G	5 μg/g or 2.5 μg/g, i.v., 8 days	
Otsuka et al. (35)	Direct inhibition	Cl-amidine	80 mg/kg, i.p., 5 days	NETs modulation significantly preserved weight body and reduced histological scores (*P* < 0.05 for all results).
Shao et al. (28)	Direct degradation	DNase I	50 ug/mice, i.v., 7 days	DNase I intervention significantly aggravated weight loss, shortened colon length, increased DAI score, exacerbated colon injury, increased intestinal vascular permeability, and increased plasma inflammatory factor levels (*P* < 0.05 for all results).
	Indirect modulation	GS	20 mg/kg, i.p., 7 days	GS inhibited DNase activity significantly reduced weight loss, increased colon length, decreased DAI score, relieved colon injury, reduced intestinal vascular leakage, while reducing plasma inflammatory factor levels (*P* < 0.05 for all results).
Li et al. (87)	Indirect modulation	Butyrate	200 mM, drink, 10 days	NETs modulation significantly preserved body weight and colon length reduced histological scores, and downregulated pro-inflammatory cytokines (IL-6, TNF-α, and IFN-γ) (*P* < 0.05 for all results).

Deoxyribonuclease (DNase); thymopentin (TP5); Staphylococcal nuclease encapsulated with calcium alginate (ALG-SNase); glycyrrhizin (Gly); Dihydromyricetin (DHM); Coptis chinensis-derived extracellular vesicle-like nanoparticles (Cc-ELNs); Bacteroides Fragilis-Derived Outer Membrane Vesicles (Bf-OMVs); i.p., intraperitoneal; i.v., intravenous; i.g., intragastric; i.r., intrarectal. Interventions are classified as direct if they specifically inhibit NETs formation or degrade existing NETs, and indirect if they exert broad anti-inflammatory or pleiotropic effects. Only direct interventions were included in the quantitative meta-analysis.

### 3.3 Risk assessment of bias

Preclinical study quality was assessed with SYRCLE (**Figure 2**). All studies had low risk for baseline characteristics, incomplete data, selective reporting, and other biases. Most reported random allocation, but only five (14–18) described the random sequence generation method. Most studies were unclear due to insufficient description. No studies reported allocation concealment, random housing, random outcome assessment, or blinding of personnel (all unclear). None of the 36 studies had a high risk in any domain. Overall, methodological quality was moderate: baseline comparability and data integrity were good, but reporting of randomization and blinding was insufficient.

**Figure 2:**
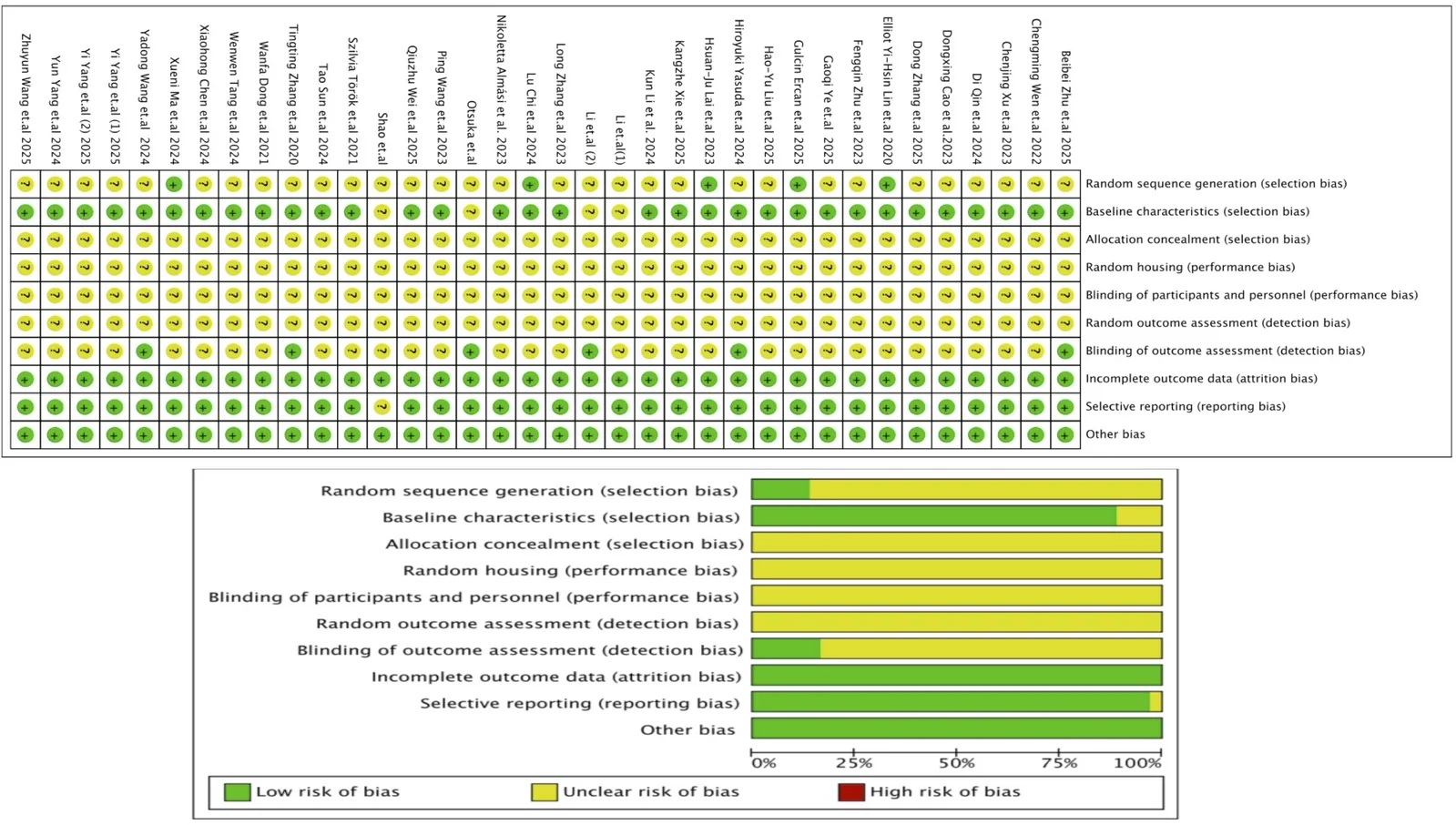
Risk of bias analysis for pre-clinical studies using the SYRCLE tool.

Clinical study quality was assessed with NOS and JBI. For the 12 case-control studies, the NOS score ranged from 7 to 9 (mean ± SD = 8.25 ± 0.97). Among the two cohort studies, Cao et al. (19) scored 8, while El Hafez et al. (20) scored 6, with deductions for lack of exposure ascertainment and inadequate follow-up. Three cross-sectional studies all met JBI criteria, except Lehmann et al. (21), which lacked clear reporting on confounding factors. Detailed assessments are in **Supplementary Tables 2**-**4**.

### 3.4 NETs-associated biomarkers in clinical IBD patients

Across all included studies, NETs-associated biomarkers were consistently elevated in IBD patients compared with healthy controls or remission-state patients, employing a range of NETs markers and analytical techniques. ELISA-based analyses revealed elevated levels of circulating MPO-DNA complexes (22–24), CitH3 (19, 23, 25–28), MPO (19) and NE (24) in the peripheral blood of IBD patients, while one study reported no significant difference in plasma CitH3 between IBD patients and healthy controls (26). IHC and IF staining consistently confirmed increased colonic deposition of MPO (19, 25, 28–31), NE (19, 22, 29–34), PAD4 (19, 20, 29), and CitH3 (19, 22, 25, 28, 29, 31–34) in inflamed mucosal areas of IBD patients compared with non-inflamed areas or healthy control tissues. Fecal metaproteomic analysis further revealed upregulation of NETs-related proteins (NE, MPO, azurocidin, cathepsin G) in IBD patients (21). Most studies did not observe significant differences in NETs-associated marker levels between UC and CD patients. For instance, in a pediatric cohort, serum NE and MPO-DNA levels were similarly elevated in both UC and CD patients relative to healthy controls, with no statistically significant difference between the two subtypes (24). Otsuka et al. (35) found that colonic mucosal PAD4 mRNA was elevated in active UC compared with UC in remission, whereas no such difference was observed between active and remission phases of CD, suggesting that PAD4-dependent NETs may be more prominently involved in UC than CD. However, direct comparison between UC and CD was not performed in most included studies, and the available data do not permit a definitive conclusion regarding disease-specific NETs profiles. Importantly, elevated NETs-associated marker levels correlated positively with disease activity and severity. Colonic PAD4 expression correlated positively with histopathological activity grade and was associated with therapy response and the need for radical surgery in UC patients (20). Mucosal PAD4 mRNA levels correlated positively with disease activity indices and pro-inflammatory cytokine profiles (35). Circulating NETs marker levels correlated positively with inflammatory factors, and a high NETs score emerged as an independent poor prognostic factor for IBD relapse (23). In patients with perianal fistulizing CD, NETs levels in fistulas correlated inversely with local infliximab concentrations and fistula healing, implicating NETs accumulation in treatment resistance (19, 25). However, one pediatric study found that while NETs-associated markers were elevated in active IBD children compared with healthy controls, they did not correlate with clinical or endoscopic activity scores (24), indicating that the associations with disease activity may vary across age groups and disease phenotypes.

### 3.5 Meta-analysis

#### 3.5.1 NETs-associated biomarkers are elevated in pre-clinical models

We performed a stratified meta-analysis of baseline NETs-associated biomarkers across five detection methods (WB, IHC, IF, ELISA, and qPCR) to assess the consistency of evidence across independent assay platforms. WB revealed increased colonic CitH3 (6.31 [4.24, 8.38], I^2^ = 85.9%, *P<*0.001), MPO (7.06 [4.11, 10.02], I^2^ = 88.5%, *P<*0.001), and PAD4 (4.39 [1.27, 7.50], I^2^ = 89.0%, *P* = 0.006). IHC and IF similarly showed upregulation of NE (1.43 [0.31, 2.56], I^2^ = 57.8%, *P* = 0.013) and Ly6G (7.31 [2.70, 11.92], I^2^ = 11.0%, *P* = 0.002). ELISA confirmed increased soluble MPO-DNA (3.31 [1.86, 4.75], I^2^ = 81.1%, *P<*0.001), cell-free CitH3 (3.08 [1.90, 4.25], I^2^ = 0.0%, *P<*0.001), and MPO (3.93 [0.45, 7.40], I^2^ = 85.4%, *P* = 0.027) in blood and feces. qPCR trends for PAD4, MPO, and ELANE were non-significant (**Figure 3**).

**Figure 3:**
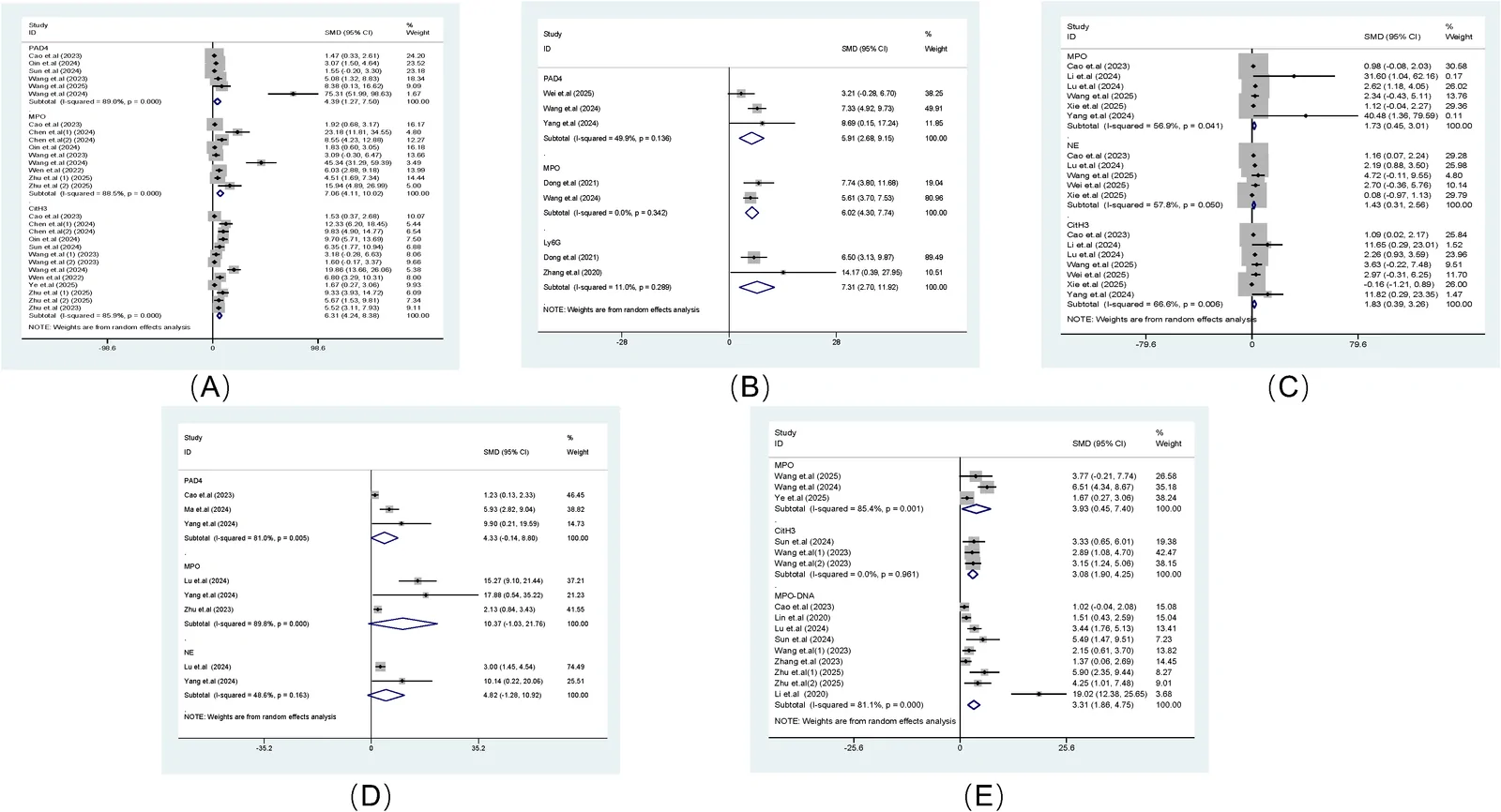
Forest plots of NETs-associated biomarkers at baseline in preclinical IBD models. Each panel displays the SMD with 95% CI for individual studies and the pooled estimate comparing IBD model groups with corresponding controls, stratified by detection method: **(A)** Western blot, **(B)** immunohistochemistry, **(C)** immunofluorescence, **(D)** quantitative PCR, and **(E)** enzyme-linked immunosorbent assay. A random-effects model was applied. The I^2^ statistic and its associated P value are reported for each meta-analysis. The size of each square reflects study weight; the diamond represents the overall pooled estimate. Enlarged single-panel versions of each forest plot are available in **Supplementary Figures 3A–E**.

Subgroup analyses were performed to explore potential sources of heterogeneity (**Supplementary Table 5**). For PCR detection of PAD4, stratification by modeling method yielded a significant subgroup difference (*Q_-_*_between_ = 9.92*, P* = 0.002). For WB detection of PAD4 (*Q_-_*_between_ = 4.22*, P* = 0.040) and of MPO (*Q_-_*_between_ = 11.23*, P* = 0.001), a similar pattern was observed between DSS and TNBS models. However, for most other comparisons, including ELISA-based MPO-DNA (*Q_-_*_between_ = 5.70*, P* = 0.058) by sample type and WB-based markers (*P* > 0.05 for all) by reference gene, the differences between subgroups were not statistically significant. Moreover, substantial within-group heterogeneity persisted in several subgroups, and the small number of studies within individual subgroups limited the statistical power of these exploratory analyses.

Leave-one-out sensitivity analysis (**Supplementary Figure 1**) showed stable core outcomes for most markers. However, PCR-PAD4 and IHC-PAD4 lost significance after excluding Cao et al. (36) and Wang et al. (6), respectively; omitting Wang et al. (6) also reduced I^2^ for IHC-PAD4 to 26.3%. Thus, these pooled results depend on single studies and need validation. Only the WB-CitH3 subgroup has more than 10 studies. Funnel plot asymmetry (**Supplementary Figure 2**) and Begg’s (*P* = 0.001) and Egger’s tests (*P<* 0.001) indicated publication bias. Trim-and-fill adjustment found no missing studies (zero imputed), and the pooled effect remained unchanged (6.31 [4.24, 8.38]).

#### 3.5.2 The reduction of NETs-associated biomarkers after modulation

To validate the mechanistic efficacy of direct NET modulation, we analyzed pooled NETs-associated biomarkers following intervention (**Supplementary Figure 3**). Targeted interventions reduced NETs-associated biomarkers in both colonic tissues and peripheral blood. In colonic tissues, WB analysis showed a significant downregulation of CitH3 (SMD=-1.00, 95% CI [-1.65, -0.34], I^2^ = 0.0%, *P* = 0.003). Pooled IF and IHC data revealed that colonic MPO (SMD=-0.82, 95% CI [-1.43, -0.20], I^2^ = 0.0%, *P* = 0.009), NE (SMD=-2.41, 95% CI [-4.37, -0.46], I^2^ = 63.9%, *P* = 0.015), and Ly6G (SMD=-5.90, 95% CI [-10.76, -1.04], I^2^ = 76.5%, *P* = 0.017) levels were significantly lower in the intervention group compared to the untreated group. Pooled ELISA data further supported this conclusion, showing a significant decrease in soluble MPO-DNA (SMD=-1.42, 95% CI [-2.60, -0.23], I^2^ = 57.9%, *P* = 0.019) complexes and CitH3 (SMD=-2.13, 95% CI [-3.22, -1.04], I^2^ = 0.0%, *P<* 0.001) in the peripheral blood. Although the reduction in some colonic protein markers (PAD4 and MPO via WB) lacked statistical significance, the pooled effect sizes showed a consistent downward trend. Overall, direct modulation strategies effectively reduce NETs burden, providing a biological basis for associated improvements in clinical phenotypes.

#### 3.5.3 Clinical and histopathological improvements following NETs modulation

The DAI, HS, and colon length are core indicators of colitis severity. Both NETs inhibition and degradation reduced DAI (inhibition: -1.00 [-1.81, -0.18], I^2^ = 67.5%, *P* = 0.017; degradation: −1.71 [-2.71, -0.71], I^2^ = 75.7%, *P* = 0.001) and histological scores (inhibition: -1.70 [-2.60, -0.81], I^2^ = 73.3%, *P<* 0.001; degradation: -2.95 [-4.73, -1.17], I^2^ = 87.4%, *P* = 0.001). Both strategies also protected colon length (degradation: 1.17 [0.32, 2.02], I^2^ = 58.9%, *P* = 0.007; inhibition: 1.27 [0.26, 2.28], I^2^ = 78.4%, *P* = 0.014) (**Figure 4**).

**Figure 4:**
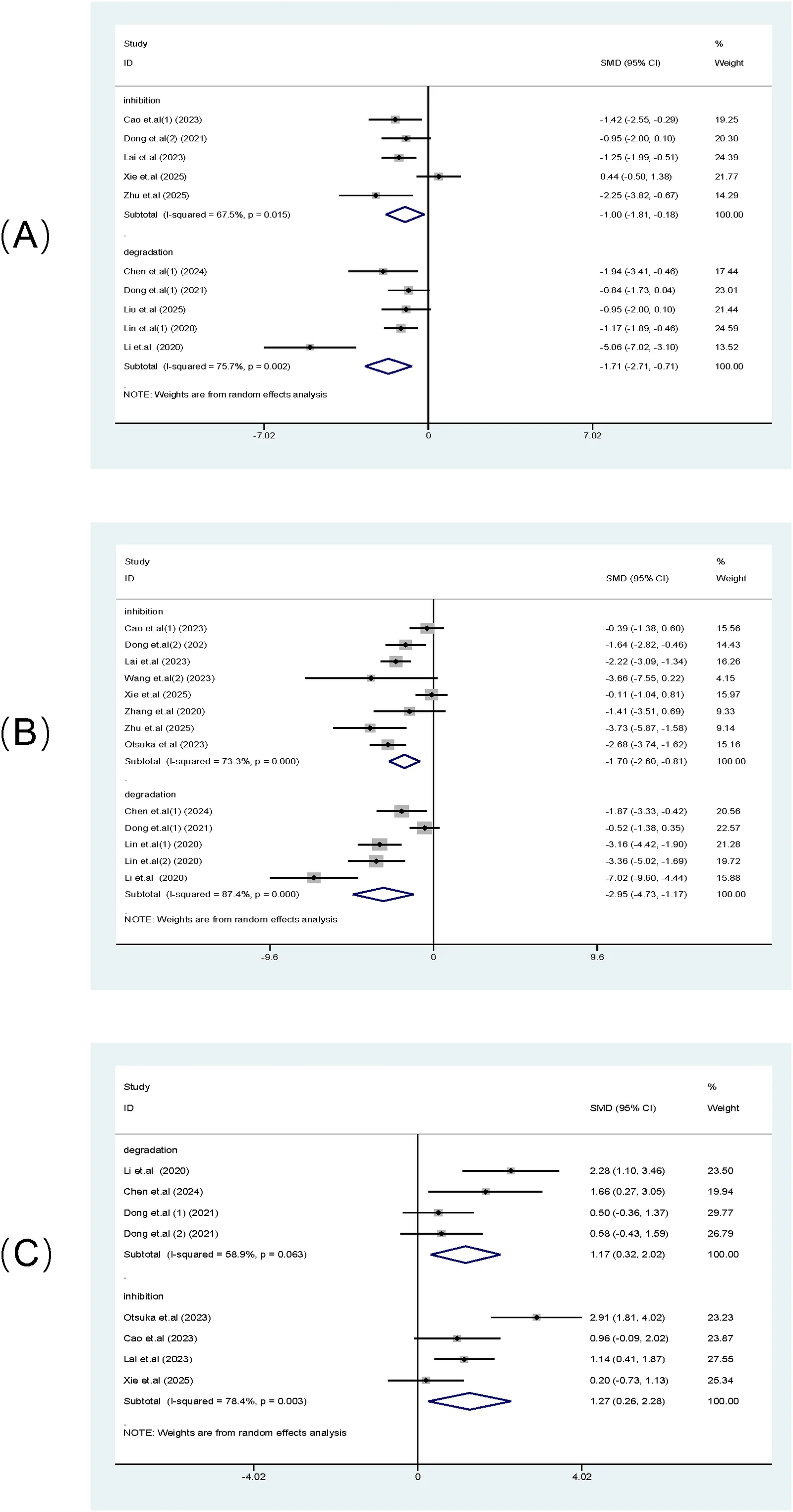
Forest plots of clinical and pathological outcomes following direct NETs modulation in preclinical IBD models. Each panel displays the SMD with 95% CI for individual studies and the pooled estimate, stratified by intervention type (inhibition versus degradation): **(A)** DAI, **(B)** HS, and **(C)** colon length. A random-effects model was applied. The I^2^ statistic and its associated P value are reported for each meta-analysis. The size of each square reflects study weight; the diamond represents the overall pooled estimate. For DAI and histological score, negative SMD values favor the intervention group, indicating reduced disease severity. For colon length, positive SMD values favor the intervention group, indicating protection against colon shortening. Enlarged single-panel versions of each forest plot are available in **Supplementary Figures 4A–C**.

Subgroup analyses suggested that administration route and modeling method may contribute to heterogeneity for some outcomes. For histological scores, administration route showed significant subgroup difference in both the inhibition group (*Q_-_*_between_ = 8.29*, P* = 0.040) and the degradation group (*Q_-_*_between_ = 10.57*, P* = 0.001). The modeling method also showed a significant subgroup difference for histological score in the inhibition group (*Q_-_*_between_ = 4.16*, P* = 0.041). For colon length, a significant subgroup difference was observed only in the degradation group by administration route (*Q_-_*_between_ = 6.85*, P* = 0.009). Of note, the number of studies within several subgroups was small, with some subgroups containing only a single study. The observed differences between subgroups should therefore be interpreted as exploratory rather than confirmatory. Detailed subgroup analysis results are presented in **Supplementary Table 6**.

Leave-one-out sensitivity analysis (**Supplementary Figure 4**) showed HS results were stable. However, DAI improvement under inhibition was unstable: excluding Xie et al. (37) increased the effect size, while omitting any other study caused a loss of significance. Colon length after inhibition was also unstable: excluding Otsuka et al. (35) shifted the estimate leftward, and removing Lai et al. (16) widened the confidence interval, losing significance. Thus, the protective effect of inhibition on colon length is driven by influential studies, and the robustness of DAI and colon length results needs validation.

#### 3.5.4 Restoration of intestinal barrier integrity following NETs modulation

FITC-dextran permeability was improved by both NETs inhibition (-0.92 [-1.72, -0.12], I^2^ = 20.9%, *P* = 0.024) and degradation (-0.81 [-1.41, -0.21], I^2^ = 0.0%, *P* = 0.008). Both strategies upregulated tight junction protein occludin (inhibition: 1.35 [0.62, 2.08], I^2^ = 0.0%, *P<* 0.001; degradation: 2.59 [0.84, 4.33], I^2^ = 66.4%, *P* = 0.004). However, ZO-1 expression showed upward trends but was not significant (inhibition: 0.86 [-0.60, 2.33], I^2^ = 72.6%, *P* = 0.249; degradation: 2.76 [-0.11, 5.63], I^2^ = 86.0%, *P* = 0.059) (**Figure 5**).

**Figure 5:**
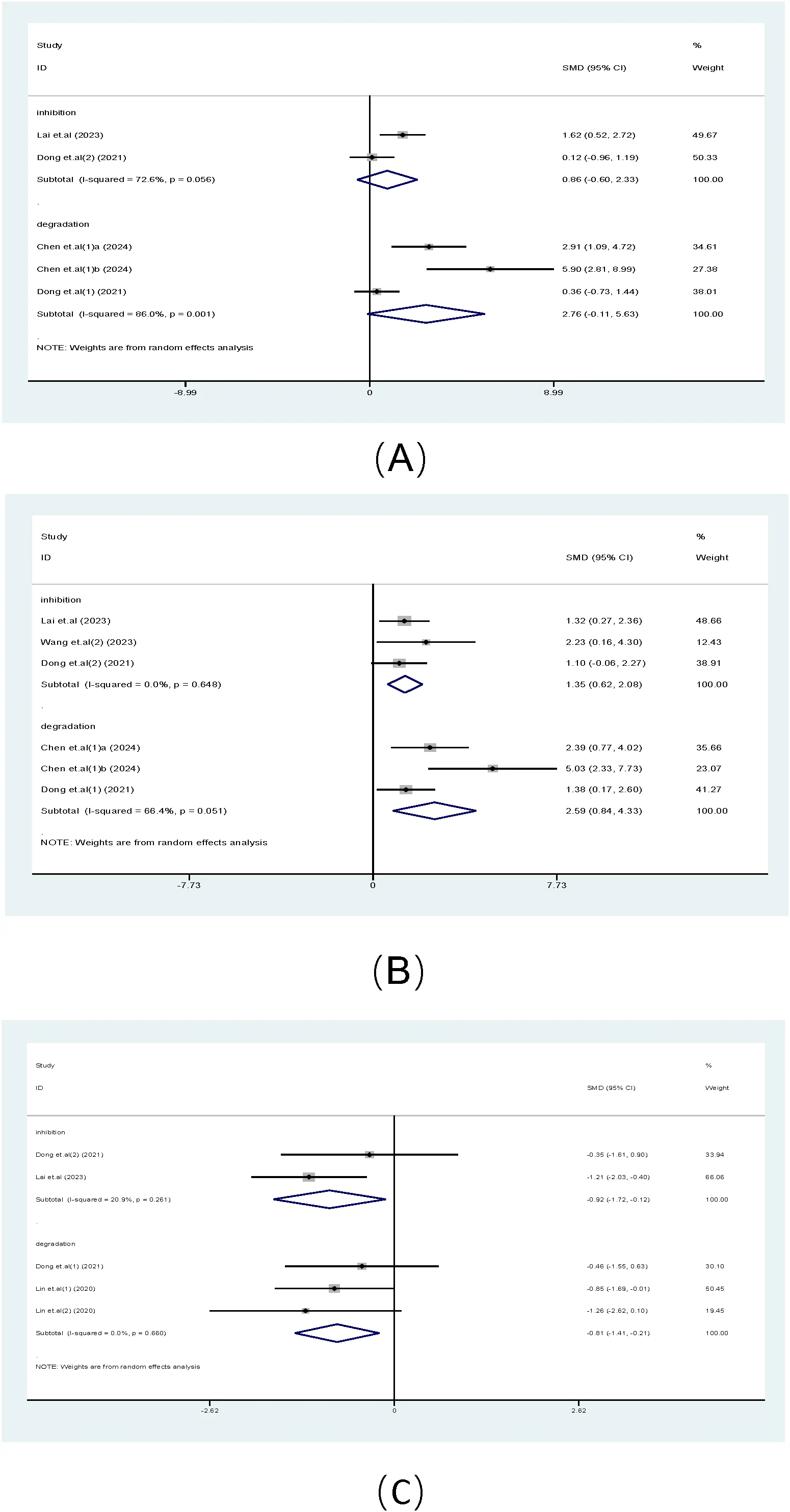
Forest plots of intestinal barrier function outcomes following direct NETs modulation in preclinical IBD models. Each panel displays the SMD with 95% CI for individual studies and the pooled estimate: **(A)** ZO-1 expression, **(B)** occludin expression, and **(C)** FITC-dextran permeability. A random-effects model was applied. The I^2^ statistic and its associated P value are reported for each meta-analysis. The size of each square reflects study weight; the diamond represents the overall pooled estimate. For ZO-1 and occludin, positive SMD values favor the intervention group, indicating restoration of tight junction integrity. For FITC-dextran permeability, negative SMD values favor the intervention group, indicating reduced intestinal permeability. Enlarged single-panel versions of each forest plot are available in **Supplementary Figures 5A–C**.

For ZO-1 in the degradation group, stratification by administration route suggested a significant subgroup difference (*Q_-_*_between_ = 5.57*, P* = 0.016). Administration route and detection method did not yield significant subgroup differences for occludin either the inhibition group or the degradation group (*P* > 0.05). However, the small number of studies within each subgroup substantially limited the reliability of these comparisons. Detailed subgroup analysis results are presented in **Supplementary Table 6**.

Leave-one-out analysis (**Supplementary Figure 5**) showed that only occludin after NETs inhibition was robust. Other indices depended on single studies. For instance, within the degradation, omitting studies by Lin et al. (14) and Chen et al. (38) eliminated significance for FITC-dextran permeability and occludin expression. For the overall analysis of ZO-1, which was not significant, removing the study by Dong et al. (39) yielded statistical significance. Thus, NETs modulation shows a protective trend, but the robustness of some outcomes needs validation.

#### 3.5.5 The reshape of intestinal inflammatory microenvironment after modulation

For pro-inflammatory cytokines, both NETs inhibition and degradation reduced IL-1β (inhibition: -1.12 [-1.52, -0.72], I^2^ = 0.0%, *P<* 0.001; degradation: -1.40 [-2.07, -0.72], I^2^ = 49.3%, *P<* 0.001), IL-6 (inhibition: -1.10 [-1.73, -0.46], I^2^ = 36.8%, *P<* 0.001; degradation: -1.44 [-2.42, -0.45], I^2^ = 0.0%, *P* = 0.004), and TNF-α (inhibition: -1.15 [-1.59, -0.72], I^2^ = 0.0%, *P<* 0.001; degradation: -1.88 [-2.56, -1.19], I^2^ = 44.7%, *P<* 0.001) with low heterogeneity. NETs degradation also reduced IFN-γ (-3.24 [-6.19, -0.29], I^2^ = 83.5%, *P* = 0.031). IL-17A showed non-significant downward trends (inhibition: -0.80 [-1.62, 0.01], I^2^ = 0.0%, *P* = 0.054; degradation: -0.75 [-1.64, 0.15], I^2^ = 65.9%, *P* = 0.101). By contrast, NETs degradation increased anti-inflammatory IL-10 (6.09 [0.03, 12.14], I^2^ = 81.7%, *P* = 0.049) and TGF-β (5.95 [3.56, 8.33], I^2^ = 10.4%, *P<* 0.001) (**Figure 6**).

**Figure 6:**
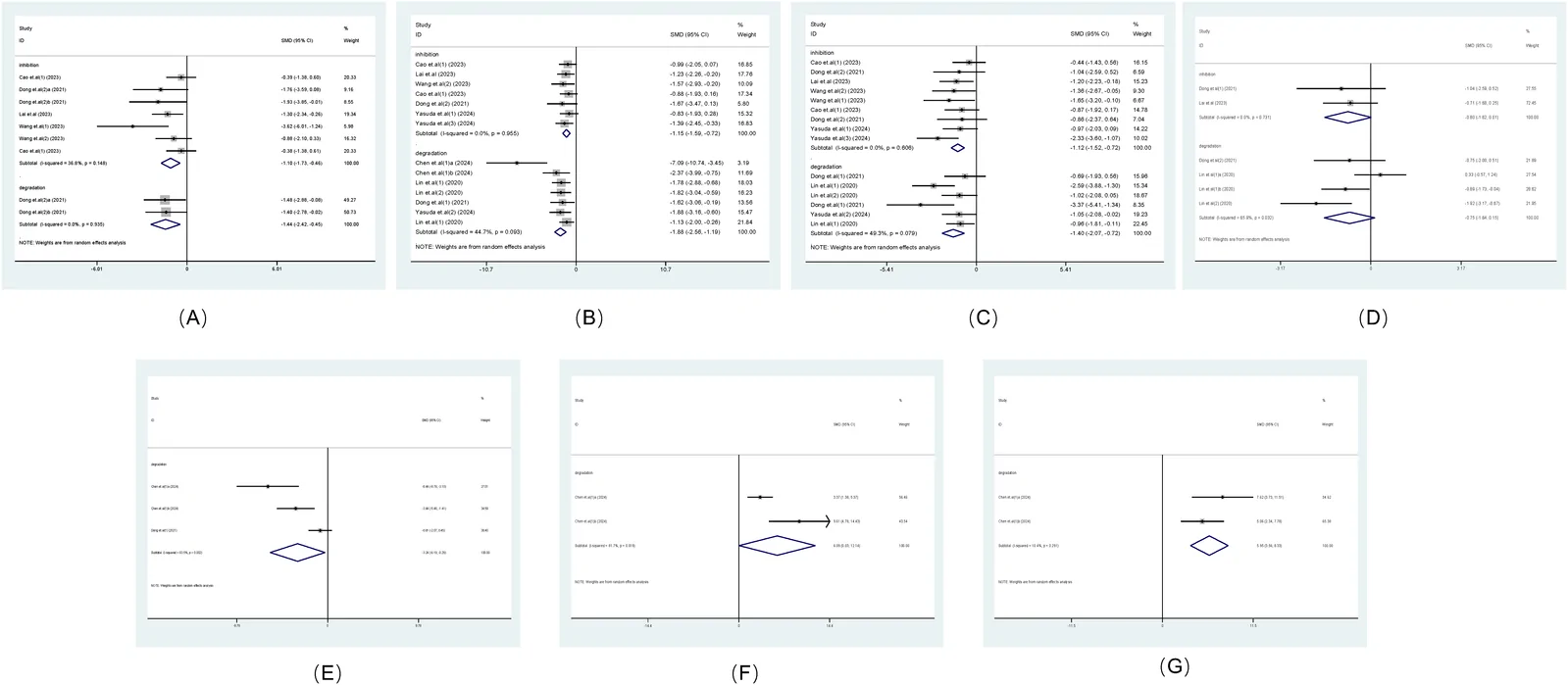
Forest plots of inflammatory cytokine expression following direct NETs modulation in preclinical IBD models. Each panel displays the SMD with 95% CI for individual studies and the pooled estimate: **(A)** IL-6, **(B)** TNF-α, **(C)** IL-1β, **(D)** IL-17A, **(E)** IFN-γ, **(F)** IL-10, and **(G)** TGF-β. A random-effects model was applied. The I^2^ statistic and its associated P value are reported for each meta-analysis. The size of each square reflects study weight; the diamond represents the overall pooled estimate. For pro-inflammatory cytokines (IL-6, TNF-α, IL-1β, IL-17A, and IFN-γ), negative SMD values favor the intervention group, indicating suppression of inflammatory responses. For anti-inflammatory cytokines (IL-10 and TGF-β), positive SMD values favor the intervention group, indicating enhanced immune regulation. Enlarged single-panel versions of each forest plot are available in the **Supplementary Figures 6A–G**.

Subgroup analyses were performed to explore potential sources of heterogeneity. For IL-6 in the inhibition group, stratification by modeling method showed a significant subgroup difference (*Q_-_*_between_ = 4.97*, P* = 0.026), and within-subgroup heterogeneity was low in both subgroups. For TNF-α in the degradation group, sample type showed a significant subgroup difference (*Q_-_*_between_ = 8.47*, P* = 0.004). For IL-17A, stratification by modeling method showed a significant subgroup difference (*Q_-_*_between_ = 3.93*, P* = 0.047). For IFN-γ in the degradation group, stratification by administration route showed a significant subgroup difference (*Q_-_*_between_ = 5.68*, P* = 0.017). The number of studies within each subgroup was generally small, with many subgroups containing only one or two studies. Although some subgroup comparisons reached statistical significance, these findings should not be overinterpreted as definitive evidence for effect modification. Detailed subgroup analysis results are presented in **Supplementary Table 7**.

Leave-one-out analysis (**Supplementary Figure 6**) confirmed stable results for IL-1β, IL-6, and TNF-α. For IL-17A, excluding Lin et al. (14) made it significant. Thus, suppression of classical pro-inflammatory cytokines is robust, but IL-17A results depend on the weight of individual studies and need further validation in future large-sample experiments.

## 4 Discussion

This is the first meta-analysis to quantitatively evaluate the therapeutic potential of targeted NETs modulation in preclinical IBD models, complemented by a descriptive synthesis of clinical observational data. In summary, clinical studies show that NETs-associated biomarkers are elevated in IBD patients across multiple tissue compartments, especially in those with active disease. In a subset of patients, higher NETs burden correlates with greater disease severity and a poorer prognosis. Nevertheless, all these findings are correlational. They establish an association between NET-associated pathological activity and IBD but cannot determine causality or directly support therapeutic efficacy. The therapeutic potential of NETs modulation must therefore be evaluated through interventional studies, which are currently limited to preclinical models. Across preclinical studies, targeting NETs either by inhibiting their formation or by degrading existing NETs consistently improved disease outcomes, including DAI, histological injury, and colon length. These benefits were accompanied by restoration of the epithelial barrier, as well as a rebalanced inflammatory microenvironment.

Intestinal mucosal neutrophil infiltration is a hallmark of active IBD. Upon sensing infection or damage, mature peripheral blood neutrophils are attracted by chemokines (e.g., CCL8, CXCL10) and pro-inflammatory cytokines (IL-1β, IL-6, TNF-α) to the infection site, where they assist host defense through phagocytosis, degranulation, and NETs release (6, 40, 41). NETs consist of a DNA backbone and active proteins (histones, NE, MPO, etc.) (42). Under physiological conditions, NETs capture and eliminate pathogens, including bacteria, fungi, and viruses, to maintain homeostasis of the gut microecology (43). However, in chronic inflammation, DAMPs and PAMPs drive excessive NETs formation (44). DNA-degrading enzyme activity is reduced in IBD patients (22, 45). Impaired NETs degradation combined with overproduction leads to pathological NETs accumulation. Our meta-analysis confirmed that NETs-associated biomarkers are elevated in the colon and blood of IBD models.

Multiple biomarkers including MPO, NE, CitH3, PAD4, Ly6G, and MPO-DNA were adopted to assess NETs levels across studies included in this meta-analysis, reflecting the biological complexity of NETs and suggesting inconsistent systemic detection of NETs in practical settings. Histones undergo citrullination during NETosis, and this reaction is catalyzed by PAD4. CitH3 loosens chromatin structure, which further promotes the release of chromatin and other nuclear contents into the extracellular space (46). As a core structural and functional biomarker, CitH3 is widely used as a surrogate marker for NET detection. PAD4, the enzyme responsible for CitH3, is also regarded as a marker for NET detection. Nevertheless, not all NET formations rely on PAD4-dependent citrullination (47). Detection limited to CitH3 or PAD4 alone may miss NETs generated through alternative pathways, and negative test results do not exclude NETs formation. MPO and NE are key enzymatic constituents of neutrophil granules and play central roles in NETs generation during NETosis. Accordingly, MPO and NE are widely used for NETs measurement (10, 48, 49). Nevertheless, recent studies have demonstrated that inhibition or deficiency of MPO or NE cannot completely abrogate NETs formation, suggesting that MPO and NE are not essential for all NETs-generating pathways and that alternative mechanisms exist (50–52). In addition to their neutrophil-specific features, MPO and NE are secreted during neutrophil degranulation (53). Detectable levels of these two molecules, therefore, cannot confirm the specific formation of NETs. Although Western blot and PCR can reveal the transcription, expression, or modification of NETs-related molecular levels, these techniques have no spatial resolution. They cannot confirm whether CitH3 integrates with extracellular DNA backbones and neutrophil granule proteins. As microscopy technologies evolve, immunofluorescence microscopy that detects the colocalization of CitH3, MPO, and NE represents a specific method for visualizing NETs structures. It offers high sensitivity and is considered the most direct evidence (54). The results of immunofluorescence staining are presented in the form of picture data; therefore, this approach tends to carry the subjective judgement of researchers and is often difficult in terms of quantification. In addition, although circulating MPO-DNA complexes are elevated in patients with IBD (42), detection of such complex markers does not provide conclusive evidence of NETs formation because free DNA from any apoptotic source can bind to neutrophil-derived proteins (46). The methods used for biomarker identification and detection in NETs investigations vary greatly. Currently, there are no specific biomarkers or assays that can accurately and consistently detect NETs (55). This increases the complexity of our meta-analysis and reduces the comparability and reliability of results across NETs-related studies, necessitating a more cautious interpretation of pooled data. To this end, we stratified our meta-analysis by different detection methods (WB, qPCR, IHC, IF, and ELISA). We evaluated whether the direction and magnitude of effect sizes were consistent across platforms, which constitutes a convergent evidence approach that avoids over-reliance on any single marker. Despite the inherent limitation of individual markers, the convergence of signal across WB, IHC, IF, and ELISA platforms supports the interpretation that NETs-associated pathological activity is elevated in pre-clinical IBD models. In the future, standardized detection methods must be developed to combine multiple NETs-related markers with highly sensitive assays are needed to improve reliability and accelerate clinical application.

Pro-inflammatory cytokines in the IBD microenvironment trigger NETosis (48). TNF-α activates the NF-κB pathway to upregulate NETs-related genes (56). Regulated by REDD1, IL-1β promotes neutrophil recruitment and autophagy-dependent NETosis (32). IL-8 mediates NETosis via the MAPK/NADPH pathway (57). NETs induce M1 macrophage polarization via the TLR9 pathway, increasing pro-inflammatory cytokines and suppressing anti-inflammatory (58). Free histones in NETs activate TLR/STAT3 signaling in naive T cells to promote Th17 differentiation (59). Accumulated NETs act as DAMPs that amplify inflammatory signals, forming a positive feedback loop. In DSS-induced colitis, NETs-derived histones and NE directly degrade tight junctions, thereby increasing permeability (14, 16). This neutrophil- driven barrier disruption is further amplified by crosstalk with macrophages. We have reported that S100A8 mediates neutrophil-macrophage communication, reducing occludin and ZO-1 expression (60). Our meta-analysis shows that targeting NETs reduces pro-inflammatory cytokines, increases anti-inflammatory mediators, and restores barrier function, confirming the core role of NETs in IBD pathogenesis.

Strategies to modulate NETs primarily involve inhibiting their formation or promoting their degradation. PAD4 is a critical enzyme driving NET formation and thus represents a core therapeutic target. The representative inhibitor Cl-amidine covalently modifies the active site of PAD4, irreversibly suppressing its catalytic activity to block NET assembly (61). GSK484, a highly selective PAD4 inhibitor, reduces NET formation in IBD models (37). Because NETosis depends on NADPH oxidase and ROS, targeting the NOX/ROS pathway offers another approach. The NADPH oxidase inhibitor diphenyleneiodonium (DPI) lowers intracellular ROS levels in neutrophils (43). Cyclosporine A (CsA) prevents NETosis by inhibiting G6PD, the rate-limiting enzyme of the pentose phosphate pathway (PPP), to reduce ROS production (34, 62). Cleaving the extracellular DNA backbones of NETs, DNase I remains the most widely used degradation agent. However, DNase I is limited by its short half-life and biological instability (28). Importantly, simply dismantling the DNA backbone may expose intact toxic components, such as histones and proteases, directly to the intestinal mucosa, paradoxically exacerbating microvascular injury (28). Prolonged systemic clearance of NETs also increases the risk of host immune dysregulation and opportunistic infections (63). Therefore, despite its therapeutic promise, the clinical translation of DNase I requires careful consideration. Distinct from single-target interventions, natural product extracts such as dihydromyricetin (18), quercetin (64), berberine (65), as well as traditional Chinese medicine formulations like Kui-Jie-Ling (66), offer multi-target synergistic advantages and have emerged as novel strategies for NETs modulation. These indirect approaches may reduce NETs-associated markers through pleiotropic mechanisms including broad anti-inflammatory effects, regulation of gut microbiota, or modulation of immune cell function, and their effects on NET cannot be unequivocally attributed to direct NETs targeting. Nevertheless, they were included in the systematic review and summarized descriptively, as they provide valuable exploratory insights and highlight the translational potential of targeting the NETs-associated inflammatory cascade through diverse pathways.

It is important to note that this study has some limitations. The substantial heterogeneity observed across our meta-analyses warrants careful consideration. Several sources may contribute to this heterogeneity. A major source is the diversity of experimental colitis models. DSS-induced acute injury models, which constituted most included studies, primarily involve innate immune responses and epithelial barrier disruption. TNBS-induced models engage adaptive immunity. The predominance of DSS models likely explains the non-significant pooled effect size and high heterogeneity of the adaptive immune marker IL-17A in our analysis. The lack of sufficient data from chronic, adaptive immunity-driven models represents a notable gap in the current evidence. Detection methods contributed another layer of variability. Currently, the most widespread issues in the field of NETosis are the pervasive reliance on unvalidated detection techniques and the absence of standard operating protocols. The evaluation of NETs-related biomarkers may be impacted by the selection of operating techniques, including antibody selection, protein extraction efficiency, sample processing, and quantitative criteria. As a result, depending on the assay employed, NETs levels in IBD models may be exaggerated or underestimated, which directly affects the assessment of therapy effects in intervention studies. Additionally, differences in dosage, treatment duration, timing of intervention relative to disease induction, and administration route are substantial. Our subgroup analysis showed robust pooled effects for intravenous and genetic interventions, whereas oral and intraperitoneal routes exhibited high heterogeneity. This variability largely results from rapid gastrointestinal degradation of orally administered macromolecular enzymes and peptides. Moreover, the physiological changes in IBD inherently hinder drug delivery. Mucosal inflammation disrupts intestinal motility, capacity, and barrier integrity, altering drug absorption and microbial metabolism. Simultaneously, prolonged small intestinal transit and accelerated colonic transit in IBD create spatiotemporal variations in drug delivery (67). These physiological changes create spatiotemporal variations in drug delivery that are difficult to control across studies. The heterogeneity associated with intervention protocols thus has a biological basis rooted in the disease process itself, rather than merely reflecting differences in experimental design. Therefore, future drug development should incorporate precision delivery technologies, such as receptor-mediated nanoparticles (68), to achieve colon-targeted release, thereby increasing local bioavailability and avoiding systemic side effects. The small number of studies within each subgroup further limited the reliability of our subgroup analyses. Many subgroups contained fewer than three studies, and the observed reductions in within-subgroup I^2^ following stratification may partly reflect insufficient statistical power rather than genuine identification of heterogeneity sources. Formal tests for subgroup differences were largely non-significant, and the small sample sizes limited the reliability of these comparisons. A fundamental limitation of the current evidence base is that the therapeutic efficacy data are derived exclusively from preclinical animal models. The observational clinical studies included in this review are correlative in nature and cannot establish causality or predict therapeutic response in humans. The clinical evidence included in this review was derived predominantly from mixed IBD cohorts or single-subtype studies, without direct head-to-head comparisons between UC and CD. The available data suggest that NETs-associated biomarker profiles may differ between the two subtypes and across disease activity states, but the small number of studies and heterogeneous assay methods precluded formal subgroup analysis. Further studies with standardized protocols and stratified analyses by disease subtype and activity are needed to clarify whether NETs accumulation exhibits disease-specific characteristics and to inform the development of subtype-targeted therapeutic strategies.

## 5 Conclusion

This systematic review confirms that NETs-associated biomarkers are elevated in IBD patients and preclinical models, supporting the pathological relevance of NETs in IBD. In preclinical studies, inhibiting or degrading NETs improves disease phenotypes, restores intestinal barrier function, and interrupts inflammatory cascades in experimental colitis models. However, current evidence is insufficient to recommend any specific NETs-targeted intervention for clinical use. The clinical studies included in this review are observational and correlative, and cannot establish causality or predict therapeutic efficacy in humans. High-quality preclinical studies with standardized protocols, alongside rigorous translational research, are needed to define optimal therapeutic parameters and delivery strategies before clinical trials can be justified.

## Data Availability

The original contributions presented in the study are included in the article/**Supplementary Material**. Further inquiries can be directed to the corresponding author/s.
