# Supplementary material for: Neutrophil extracellular traps as a potential therapeutic target in inflammatory bowel disease: a systematic review and meta-analysis
Source: Front Immunol. 2026 Sep 17;17:1935261. doi: 10.3389/fimmu.2026.1935261 (PMC13627307; doi:10.3389/fimmu.2026.1935261)
Supplement: Supplementary file 1 [file Table1.docx]

| **Supplementary Table 1. Search strategy on PubMed** | |
| --- | --- |
| #1 | ("Extracellular Traps"[Mesh]) OR ((((((((((((((((((((((((((Extracellular Trap) OR (Trap, Extracellular)) OR (Traps, Extracellular)) OR (Extracellular DNA Traps)) OR (DNA Trap, Extracellular)) OR (DNA Traps, Extracellular)) OR (Extracellular DNA Trap)) OR (Trap, Extracellular DNA)) OR (Traps, Extracellular DNA)) OR (Neutrophil Extracellular Traps)) OR (Extracellular Trap, Neutrophil)) OR (Extracellular Traps, Neutrophil)) OR (Neutrophil Extracellular Trap)) OR (Trap, Neutrophil Extracellular)) OR (Traps, Neutrophil Extracellular)) OR (NETs (Neutrophil Extracellular Traps))) OR (NET (Neutrophil Extracellular Traps))) OR (Eosinophil Extracellular Traps)) OR (Eosinophil Extracellular Trap)) OR (Extracellular Trap, Eosinophil)) OR (Extracellular Traps, Eosinophil)) OR (Trap, Eosinophil Extracellular)) OR (Traps, Eosinophil Extracellular)) OR (EEDTs (Eosinophil Extracellular DNA Traps))) OR (EEDT (Eosinophil Extracellular DNA Traps))) OR (Eosinophil Extracellular DNA Traps)) |
| #2 | ("Inflammatory Bowel Diseases"[Mesh]) OR ((Inflammatory Bowel Disease) OR (Bowel Diseases, Inflammatory)) |
| #3 | ("Colitis, Ulcerative"[Mesh]) OR ((((Colitis Gravis) OR (Idiopathic Proctocolitis)) OR (Inflammatory Bowel Disease, Ulcerative Colitis Type)) OR (Ulcerative Colitis)) |
| #4 | (((((((((((((((((Crohn's Disease) OR (Crohns Disease)) OR (Crohn's Enteritis)) OR (Inflammatory Bowel Disease 1)) OR (Regional Enteritis)) OR (Ileocolitis)) OR (Ileitis, Terminal)) OR (Terminal Ileitis)) OR (Ileitis, Regional)) OR (Regional Ileitides)) OR (Regional Ileitis)) OR (Enteritis, Granulomatous)) OR (Granulomatous Enteritis)) OR (Enteritis, Regional)) OR (Colitis, Granulomatous)) OR (Granulomatous Colitis))) OR ("Crohn Disease"[Mesh]) |
| #5 | #2 OR #3 OR #4 |
| #6 | #1 AND #5 |

| **Supplementary Table 2: The Newcastle-Ottawa Scale (NOS) for case-control studies** | | | | | | | | | |
| --- | --- | --- | --- | --- | --- | --- | --- | --- | --- |
| Study | Selection | | | | Comparability | Outcome | | | |
|  | Is the case definition adequate? | Representativeness of the cases | Selection of controls | Definition of controls | Comparability of cases and controls based on the design or analysis | Ascertainment of exposure | Same method of ascertainment for cases and controls | Non-response rate | Score |
| Angelidou et al. | 1 | 1 | 1 | 1 | 2 | 1 | 1 | 1 | 9 |
| Dinallo et al. | 1 | 1 | 1 | 1 | 2 | 1 | 1 | 1 | 9 |
| Drury et al. | 1 | 1 | 1 | 1 | 2 | 1 | 1 | 1 | 9 |
| Gottlieb et al. | 1 | 1 | 1 | 1 | 1 | 1 | 1 | 1 | 8 |
| Li et al. | 1 | 1 | 1 | 1 | 2 | 1 | 1 | 1 | 9 |
| Lu et al. | 1 | 1 | 1 | 1 | 2 | 1 | 1 | 1 | 9 |
| Otsuka et al. | 1 | 1 | 1 | 1 | 2 | 1 | 1 | 1 | 9 |
| Schoen et al. | 1 | 1 | 1 | 1 | 2 | 1 | 1 | 1 | 9 |
| Schroder et al. | 1 | 1 | 0 | 1 | 1 | 1 | 1 | 1 | 7 |
| Shao et al. | 1 | 1 | 0 | 1 | 1 | 1 | 1 | 1 | 7 |
| Shukrun et al. | 1 | 1 | 0 | 1 | 1 | 1 | 1 | 1 | 7 |
| Li et al. | 1 | 1 | 0 | 1 | 1 | 1 | 1 | 1 | 7 |

| **Supplementary Table 3: The Newcastle-Ottawa Scale (NOS) for Cohort studies** | | | | | | | | | |
| --- | --- | --- | --- | --- | --- | --- | --- | --- | --- |
| Study | Selection | | | | Comparability | Outcome | | | |
|  | Demonstration that outcome of interest was not present at start of study | Representativeness of the exposed cohort | Selection of the non-exposed cohort | Ascertainment of exposure | Comparability of cohorts based on the design or analysis controlled for confounders | Assessment of outcome | Was follow-up long enough for outcomes to occur | Adequacy of follow-up of cohorts | Score |
| El Hafez et al. | 1 | 1 | 1 | 0 | 1 | 1 | 1 | 0 | 6 |
| Cao et al. | 1 | 1 | 1 | 1 | 1 | 1 | 1 | 1 | 8 |

| **Supplementary Table 4: The JBI critical appraisal for cross-sectional studies** | | | | | | | | | | | | |
| --- | --- | --- | --- | --- | --- | --- | --- | --- | --- | --- | --- | --- |
|  | Cao et al. | | | | Lehmann et al. | | | | Kong et al. | | | |
|  | Yes | NO | Unclear | NA | Yes | NO | Unclear | NA | Yes | NO | Unclear | NA |
| Were the criteria for inclusion in the sample  clearly defined? | * |  |  |  | * |  |  |  | * |  |  |  |
| Were the study subjects and the setting described  in detail? | * |  |  |  | * |  |  |  | * |  |  |  |
| Was the exposure measured in a valid and  reliable way? | * |  |  |  | * |  |  |  | * |  |  |  |
| Were objective, standard criteria used for  measurement of the condition? | * |  |  |  | * |  |  |  | * |  |  |  |
| Were confounding factors identified? | * |  |  |  |  |  | * |  | * |  |  |  |
| Were strategies to deal with confounding factors  stated? | * |  |  |  |  |  | * |  | * |  |  |  |
| Were the outcomes measured in a valid and  reliable way? | * |  |  |  | * |  |  |  | * |  |  |  |
| Was appropriate statistical analysis used? | * |  |  |  | * |  |  |  | * |  |  |  |
| Overall appraisal | Included | | | | Included | | | | Included | | | |
| NA, not applicable. Maximum score:8 points | | | | | | | | | | | | |

| **Supplementary Table 5: Subgroup analysis of each NET marker at the baseline level** | | | | | | | | | |
| --- | --- | --- | --- | --- | --- | --- | --- | --- | --- |
| Detection method | Marker | Subgroup | | Included studies | Heterogeneity | | Z-value | P-value | SMD (95% CI) |
|  |  |  |  |  | I^2^ | P-value |  |  |  |
| qPCR | PAD4 | Modeling methods | DSS | 2 | 0.0% | *P* =0.445 | 4.17 | *P*<0.001 | 6.30 (3.34,9.26) |
|  |  |  | TNBS | 1 | / | / | 2.20 | *P*=0.028 | 1.23 (0.13,2.33) |
|  | MPO | Modeling methods | DSS | 2 | 68.3% | *P* =0.076 | 1.01 | *P*=0.313 | 7.54 (-7.12,22.19) |
|  |  |  | TNBS | 1 | / | / | 4.85 | *P*<0.001 | 15.27 (9.10,21.44) |
|  |  | Animal species | Mice | 2 | 68.3% | *P* =0.076 | 1.01 | *P*=0.313 | 7.54 (-7.117,22.189) |
|  |  |  | Rat | 1 | / | / | 4.85 | *P*<0.001 | 15.27 (9.10,21.44) |
| WB | PAD4 | Modeling methods | DSS | 5 | 90.6% | *P* <0.001 | 2.71 | *P*=0.007 | 6.64 (1.84,11.44) |
|  |  |  | TNBS | 1 | / | / | 2.52 | *P*=0.012 | 1.47 (0.33,2.61) |
|  |  | Reference gene | β-actin | 4 | 93.0% | *P*<0.001 | 2.52 | *P*=0.012 | 6.77 (1.50,12.03) |
|  |  |  | GAPDH | 2 | 64.0% | *P* =0.096 | 1.70 | *P*=0.090 | 2.91 (-0.45,6.27) |
|  | MPO | Modeling methods | DSS | 8 | 89.4% | *P* <0.001 | 4.41 | *P*<0.001 | 9.30 (5.17,13.43) |
|  |  |  | TNBS | 1 | / | / | 3.03 | *P*=0.002 | 1.92 (0.68,3.17) |
|  |  | Animal species | Mice | 8 | 89.3% | *P* <0.001 | 4.42 | *P*<0.001 | 7.36 (4.10,10.63) |
|  |  |  | Rat | 1 | / | / | 3.75 | *P*<0.001 | 6.03 (2.88,9.18) |
|  |  | Reference gene | β- actin | 3 | 94.5% | *P*<0.001 | 2.43 | *P*=0.015 | 6.09 (1.17,11.00) |
|  |  |  | GAPDH | 6 | 70.7% | *P* =0.004 | 4.25 | *P*<0.001 | 7.38 (3.98,10.78) |
|  | CitH3 | Reference gene | β- actin | 4 | 93.6% | *P* <0.001 | 3.18 | *P*=0.001 | 6.82 (2.62,11.02) |
|  |  |  | GAPDH | 9 | 71.4% | *P* <0.001 | 5.37 | *P*<0.001 | 6.12 (3.89,8.36) |
| IF | MPO | Modeling methods | DSS | 4 | 63.7% | *P* =0.041 | 1.29 | *P*=0.197 | 2.41 (-1.25,6.08) |
|  |  |  | TNBS | 2 | 69.3% | *P* =0.071 | 2.11 | *P*=0.035 | 1.72 (0.12,3.32) |
|  |  | Animal species | Mice | 5 | 53.0% | *P*= 0.075 | 1.84 | *P*=0.066 | 1.43 (-0.09,2.95) |
|  |  |  | Rat | 1 | / | / | 3.58 | *P*<0.001 | 2.62 (1.19,4.05) |
|  | NE | Modeling methods | DSS | 3 | 63.8% | *P* =0.063 | 1.33 | *P*=0.185 | 1.80 (-0.86,4.46) |
|  |  |  | TNBS | 2 | 29.5% | *P*= 0.234 | 3.13 | *P*=0.002 | 1.60 (0.60,2.61) |
|  |  | Animal species | Mice | 4 | 51.8% | *P* =0.101 | 1.76 | *P*=0.079 | 1.15 (-0.13,2.43) |
|  |  |  | Rat | 1 | / | / | 3.27 | *P*=0.001 | 2.19 (0.88,3.50) |
|  | CitH3 | Modeling methods | DSS | 5 | 70.6% | *P* =0.009 | 1.90 | *P*=0.057 | 3.25 (-0.10,6.60) |
|  |  |  | TNBS | 2 | 44.0% | *P* =0.181 | 2.78 | *P*=0.005 | 1.61 (0.47,2.74) |
|  |  | Animal species | Mice | 6 | 64.9% | *P*= 0.014 | 2.00 | *P*=0.045 | 1.79 (0.04,3.54) |
|  |  |  | Rat | 1 | / | / | 3.33 | *P*=0.001 | 2.26 (0.93,3.60) |
| ELISA | MPO-DNA | Modeling methods | DSS | 7 | 83.4% | *P* <0.001 | 3.97 | *P*<0.001 | 4.08 (2.07，6.10) |
|  |  |  | TNBS | 2 | 82.4% | *P* =0.017 | 1.77 | *P*=0.077 | 2.14 (-0.23，4.51) |
|  |  | Animal species | Mice | 8 | 82.2% | *P* <0.001 | 4.08 | *P*<0.001 | 3.35 (1.74,4.96) |
|  |  |  | Rat | 1 | / | / | 4.00 | *P*<0.001 | 3.44 (1.76,5.13) |
|  |  | Sample source | Serum | 3 | 30.5% | *P* =0.237 | 4.26 | *P*<0.001 | 3.07 (1.66,4.48) |
|  |  |  | Plasma | 5 | 88.8% | *P* <0.001 | 3.14 | *P*=0.002 | 4.67 (1.75,7.58) |
|  |  |  | Colon tissue | 1 | / | / | 2.75 | *P*=0.006 | 1.51 (0.43,2.59) |

| **Supplementary Table 6: Subgroup analysis of key indicators after modulating NETs** | | | | | | | | | |
| --- | --- | --- | --- | --- | --- | --- | --- | --- | --- |
| Key indicators | NETs modulation | Subgroup | | Included studies | Heterogeneity | | Z-value |  | SMD (95% CI) |
|  |  |  |  |  | I^2^ | P-value |  |  |  |
| DAI | Inhibition | Modeling methods | DSS | 4 | 73.7% | *P*= 0.010 | 1.78 | *P* = 0.075 | -0.91 (-1.91,0.09) |
|  |  |  | TNBS | 1 | / | / | 2.46 | *P* = 0.014 | -1.42 (-2.55, -0.29) |
|  |  | Medication | Subcutaneous injection | 1 | / | / | 2.46 | *P* = 0.014 | -1.42 (-2.55, -0.29) |
|  |  |  | oral administration | 1 | / | / | 1.76 | *P* = 0.078 | -0.95 (-2.00,0.11) |
|  |  |  | PAD4 knockout | 1 | / | / | 3.30 | *P* = 0.001 | -1.25 (-2.00, -0.51) |
|  |  |  | intraperitoneal injection | 2 | 87.9% | *P*=0.004 | 0.62 | *P* = 0.538 | -0.83 (-3.45,1.80) |
|  | Degradation | Modeling methods | DSS | 4 | 81.4% | *P*=0.001 | 2.67 | *P* = 0.008 | -1.99(-3.45, -0.53) |
|  |  |  | TNBS | 1 | / | / | 3.22 | *P* = 0.001 | -1.17(-1.89, -0.46) |
|  |  | Medication | oral administration | 1 | / | / | 1.86 | *P* = 0.062 | -0.84(-1.73,0.04) |
|  |  |  | intraperitoneal injection | 2 | 12.1% | *P*=0.286 | 2.76 | *P* = 0.006 | -1.30(-2.23, -0.38) |
|  |  |  | intravenous injection | 2 | 92.5% | *P*<0.001 | 1.55 | *P* = 0.121 | -3.00(-6.81,0.80) |
| HS | Inhibition | Modeling methods | DSS | 6 | 75.4% | *P*= 0.001 | 3.72 | *P*< 0.001 | -2.03（-3.10，-0.96） |
|  |  |  | TNBS | 2 | 0.0% | *P*= 0.389 | 1.25 | *P* = 0.210 | -0.57 (-1.47,0.32） |
|  |  | Medication | Subcutaneous injection | 1 | / | / | 0.77 | *P* = 0.443 | -0.39 (-1.38,0.60) |
|  |  |  | oral administration | 1 | / | / | 2.73 | *P* = 0.006 | -1.64 (-2.82, -0.46) |
|  |  |  | PAD4 knockout | 2 | 0.0% | *P*=0.475 | 5.25 | *P* <0.001 | -2.29 (-3.14, -1.43) |
|  |  |  | intraperitoneal injection | 4 | 82.9% | *P*=0.001 | 2.17 | *P* = 0.030 | -1.88（-3.58，-0.18) |
|  | Degradation | Modeling methods | DSS | 4 | 89.5% | *P*<0.001 | 2.66 | *P* = 0.008 | -2.88（-5.01，-0.76） |
|  |  |  | TNBS | 1 | / | / | 3.95 | *P* <0.001 | -3.36 (-5.02, -1.70) |
|  |  | Medication | oral administration | 1 | / | / | 1.17 | *P* = 0.240 | -0.52 (-1.38,0.35) |
|  |  |  | intravenous injection | 4 | 74.2% | *P*=0.009 | 4.30 | *P* <0.001 | -3.58 (-5.20, -1.95) |
| Length of the colon | Inhibition | Modeling methods | DSS | 3 | 85.4% | *P*=0.001 | 1.96 | *P* = 0.049 | 1.38（0.00，2.76） |
|  |  |  | TNBS | 1 | / | / | 1.80 | *P* = 0.072 | 0.97（-0.09，2.02） |
|  |  | Medication | Subcutaneous injection | 1 | / | / | 1.80 | *P* = 0.072 | 0.97（-0.09，2.02） |
|  |  |  | PAD4 knockout | 1 | / | / | 3.05 | *P* = 0.002 | 1.14（0.41，1.87） |
|  |  |  | intraperitoneal injection | 2 | 92.6% | *P*<0.001 | 1.13 | *P* = 0.257 | 1.54（-1.12，4.20） |
|  | Degradation | Medication | oral administration | 2 | 0.0% | *P*=0.909 | 1.60 | *P* = 0.109 | 0.54（-0.12，1.19） |
|  |  |  | intravenous injection | 2 | 0.0% | *P*=0.505 | 4.41 | *P* <0.001 | 2.02（1.12，2.90） |
| ZO-1 | Degradation | Detection method | WB | 1 | / | / | 3.14 | *P* = 0.002 | 2.91 (1.09,4.73) |
|  |  |  | IHC | 2 | 90.9% | *P*=0.001 | 1.06 | *P* = 0.289 | 2.93 (-2.49,8.35） |
|  |  | Medication | oral administration | 1 | / | / | 0.65 | *P* = 0.517 | 0.36 (-0.73,1.44) |
|  |  |  | intravenous injection | 2 | 62.5% | *P*=0.102 | 2.81 | *P* = 0.005 | 4.13 (1.25,7.01) |
| Occludin | Inhibition | Detection method | WB | 2 | 0.0% | *P*= 0.441 | 3.16 | *P* = 0.002 | 1.50 (0.57,2.44) |
|  |  |  | IHC | 1 | / | / | 1.85 | *P* = 0.064 | 1.10 (-0.06,2.27) |
|  |  | Medication | PAD4 knockout | 1 | / | / | 1.85 | *P* = 0.064 | 1.10 (-0.06,2.27) |
|  |  |  | intravenous injection | 2 | 0.0% | *P*=0.441 | 3.16 | *P* = 0.002 | 1.50 (0.57,2.44) |
|  | Degradation | Detection method | WB | 1 | / | / | 2.88 | *P* = 0.004 | 2.39 (0.77,4.02) |
|  |  |  | IHC | 2 | 82.8% | *P*=0.016 | 1.66 | *P* = 0.098 | 3.00 (-0.55,6.55) |
|  |  | Medication | oral administration | 1 | / | / | 2.23 | *P* = 0.026 | 1.39 (0.17,2.60) |
|  |  |  | intravenous injection | 2 | 62.7% | *P*=0.101 | 2.68 | *P* = 0.007 | 3.48 (0.94,6.03) |

| **Supplementary Table 7: Subgroup analysis of inflammatory factors after modulation of NETs** | | | | | | | | | |
| --- | --- | --- | --- | --- | --- | --- | --- | --- | --- |
| Marker | NETs modulation | Subgroup | | Included studies | Heterogeneity | | Z-value |  | SMD (95% CI) |
|  |  |  |  |  | I^2^ | p-value |  |  |  |
| IL-6 | Inhibition | Modeling methods | DSS | 5 | 9.9% | *P*= 0.350 | 4.22 | *P*<0.001 | -1.52 (-2.22, -0.81) |
|  |  |  | TNBS | 2 | 0.0% | *P*= 0.995 | 1.08 | *P*=0.282 | -0.39 (-1.09,0.32) |
|  |  | Medication | subcutaneous injection | 2 | 0.0% | *P*=0.995 | 1.08 | *P*=0.282 | -0.39 (-1.09,0.32) |
|  |  |  | oral administration | 3 | 0.0% | *P*= 0.437 | 3.82 | *P*<0.001 | -2.26 (-3.42, -1.10) |
|  |  |  | PAD4 knockout | 2 | 0.0% | *P*= 0.610 | 2.79 | *P*=0.005 | -1.12 (-1.91, -0.33) |
|  |  | Sample source | serum | 1 | / | / | 1.97 | *P*=0.049 | -1.93 (-3.85, -0.01) |
|  |  |  | Colon tissue | 6 | 41.0% | *P*= 0.132 | 2.98 | *P*=0.003 | -1.02(-1.70, -0.35) |
|  |  | Detection method | PCR | 2 | 39.4% | *P*= 0.199 | 1.31 | *P*=0.191 | -0.84 (-2.11,0.42) |
|  |  |  | ELISA | 5 | 45.1% | *P*=0.122 | 2.99 | *P*=0.003 | -1.25 (-2.07, -0.43) |
| TNF-α | Degradation | Modeling methods | DSS | 5 | 62.8% | *P*= 0.030 | 3.81 | *P*<0.001 | -2.03 (-3.07, -0.99) |
|  |  |  | TNBS | 2 | 0.0% | *P*= 0.946 | 4.09 | *P*<0.001 | -1.85(-2.73, -0.96) |
|  |  | Sample source | Colon tissue | 6 | 0.0% | *P*= 0.796 | 6.63 | *P*<0.001 | -1.64(-2.12, -1.15) |
|  |  |  | serum | 1 | / | / | 3.82 | *P*<0.001 | -7.10(-10.74, -3.45) |
|  |  | Detection method | ELISA | 4 | 61.8% | *P*=0.049 | 3.75 | *P*<0.001 | -2.46(-3.74, -1.18) |
|  |  |  | PCR | 3 | 0.0% | *P*=0.607 | 4.31 | *P*<0.001 | -1.42(-2.06, -0.77) |
| IL-1β | Degradation | Modeling methods | DSS | 4 | 67.1% | *P*= 0.028 | 3.01 | *P*=0.003 | -1.71 (-2.83, -0.60) |
|  |  |  | TNBS | 2 | 0.0% | *P*= 0.965 | 2.73 | *P*=0.006 | -1.03(-1.78, -0.29) |
|  |  | Sample source | Colon tissue | 5 | 54.7% | *P*=0.065 | 3.91 | *P*<0.001 | -1.55 (-2.33, -0.77) |
|  |  |  | serum | 1 | / | / | 1.08 | *P*=0.279 | -0.69 (-1.93,0.56) |
|  |  | Detection method | ELISA | 3 | 60.1% | *P*=0.081 | 2.51 | *P*=0.012 | -1.40(-2.50, -0.31) |
|  |  |  | PCR | 3 | 57.9% | *P*= 0.093 | 2.66 | *P*=0.008 | -1.45(-2.51, -0.38) |
| IL-17A | Degradation | Modeling methods | DSS | 3 | 50.9% | *P*= 0.130 | 0.99 | *P*=0.321 | -0.41 (-1.22,0.40) |
|  |  |  | TNBS | 1 | / | / | 3.01 | *P*=0.003 | -1.92 (-3.17, -0.67) |
|  |  | Sample source | Colon tissue | 3 | 77.2% | *P*=0.012 | 1.26 | *P*=0.208 | -0.77 (-1.96, 0.43) |
|  |  |  | serum | 1 | / | / | 1.17 | *P*=0.244 | -0.75 (-2.00, 0.51) |
|  |  | Detection method | ELISA | 3 | 76.0% | *P*= 0.016 | 1.06 | *P*=0.287 | -0.72(-2.06, 0.61) |
|  |  |  | PCR | 1 | / | / | 2.06 | *P*=0.040 | -0.89(-1.73, -0.04) |
| IFN-γ | Degradation | Sample source | Colon tissue | 2 | 89.5% | *P*= 0.002 | 3.33 | *P*=0.001 | -3.437(5.461,1.413) |
|  |  |  | serum | 1 | / | / | 1.21 | *P*=0.225 | -3.40(-8.90, 2.10) |
|  |  | Medication | oral administration | 1 | / | / | 1.26 | *P*=0.209 | -0.81(-2.07, 0.45) |
|  |  |  | intravenous injection | 2 | 55.9% | *P*= 0.132 | 3.15 | *P*=0.002 | -4.63(-7.51, -1.75) |

Supplementary Figure 1 Sensitivity analysis for the baseline formation of NET-associated biomarkers in pre-clinical IBD models. (A) WB for CitH3; (B) WB for PAD4; (C) ELISA for CitH3; (D) ELISA for MPO; (E) ELISA for MPO-DNA; (F) IF for CitH3; (G) IF for MPO; (H) IF for NE; (I) IHC for PAD4; (J) PCR for MPO; (K) PCR for PAD4; (L) WB for MPO

Supplementary Figure 2 Funnel plot for the baseline level of CitH3 in the WB layer

Supplementary Figure 3 Forest plot evaluating NETs-associated biomarkers after direct modulation in pre-clinical IBD models. The accumulation of NETs after direct intervention was assessed and stratified by diverse detection methodologies: (A) WB; (B) IF; (C) ELISA-inhibition; (D) ELISA-degradation; (E) IHC

Supplementary Figure 4 Sensitivity analysis of the pooled effect sizes for DAI, HS, and colon length following NETs modulation. (A) degradation subgroup for DAI; (B) inhibition subgroup for DAI; (C) degradation subgroup for HS; (D) inhibition subgroup for HS; (E) degradation subgroup for colon length; (F) degradation subgroup for colon length

Supplementary Figure 5 Sensitivity analysis of the pooled effect sizes for intestinal barrier function following NETs modulation. (A) degradation subgroup for ZO-1; (B) inhibition subgroup for occludin; (C) degradation subgroup for occluding; (D) degradation subgroup for FITC-dextran permeability

Supplementary Figure 6 Sensitivity analysis of the pooled effect sizes for inflammatory factors following NETs modulation. (A) inhibition subgroup for IL-6; (B) inhibition subgroup for TNF-α; (C) degradation subgroup for TNF-α; (D) inhibition subgroup for IL-1β; (E) degradation subgroup for IL-1β; (F) degradation subgroup for IL-17A; (G) degradation subgroup for IFN-γ
